# Optimized motion‐insensitive PDFF mapping of the liver

**DOI:** 10.1002/mrm.70047

**Published:** 2025-09-02

**Authors:** Jiayi Tang, Daiki Tamada, Raphael do Vale Souza, Aaron Faacks, Jitka Starekova, Julius F. Heidenreich, Lukas Müller, Garrett C. Fullerton, Collin J. Buelo, Jeff Kammerman, Jean H. Brittain, Scott B. Reeder, Diego Hernando

**Affiliations:** ^1^ Medical Physics University of Wisconsin‐Madison Madison Wisconsin USA; ^2^ Radiology University of Wisconsin‐Madison Madison Wisconsin USA; ^3^ Department of Diagnostic and Interventional Radiology University Hospital Würzburg Würzburg Germany; ^4^ Department of Diagnostic and Interventional Radiology University Medical Center Mainz Mainz Germany; ^5^ Calimetrix, LLC Madison Wisconsin USA; ^6^ Biomedical Engineering University of Wisconsin‐Madison Madison Wisconsin USA; ^7^ Medicine University of Wisconsin‐Madison Madison Wisconsin USA; ^8^ Emergency Medicine University of Wisconsin‐Madison Madison Wisconsin USA

**Keywords:** fat quantification, liver, MASLD, motion robustness, PDFF, proton density fat fraction

## Abstract

**Purpose:**

To implement, optimize, and validate parallel imaging (PI)‐accelerated, 2D, flip angle modulated (FAM) chemical shift‐encoded quantification of liver proton‐density fat fraction (PDFF), with motion insensitivity.

**Methods:**

The optimization cost function that determines flip angles in FAM was generalized for PI. Phantom studies and prospective studies in volunteers with varying liver fat levels were performed. Free‐breathing FAM was acquired in the axial, sagittal, and coronal planes, with varying nominal PI acceleration factors (*R*) of 1.0 to 3.0. A breath‐held, commercially available 3D chemical shift‐encoded method was acquired as reference for PDFF. Overall image quality, qualitative SNR, and motion artifacts for all methods were Likert‐scale rated. PDFF measured by FAM was compared to reference to assess bias. Test–retest repeatability was assessed for all methods by repeating acquisitions after volunteer repositioning. Noise performance was assessed with standard deviation of PDFF maps as *R* increased.

**Results:**

The reader study (*N* = 3 readers/10 subjects) demonstrated excellent image quality for FAM during free‐breathing, with reduced motion artifacts compared to breath‐held reference (*p* < 0.01). PI‐accelerated FAM shows fewer motion artifacts than unaccelerated FAM (*p* < 0.01). In all planes and accelerations, PDFF measured by FAM showed good agreement with reference PDFF measurements (mean bias: −0.4% to 2.0% PDFF; 95% limits of agreement: 2.8% to 4.0% PDFF). FAM in axial and coronal planes showed similar or improved repeatability (repeatability coefficient = 1.7% to 2.6% PDFF) compared to the reference (2.7%). Sagittal FAM shows similar or worse repeatability (repeatability coefficient = 3.0% to 3.6%). FAM with *R* = 2.0 has good noise performance and high SNR efficiency.

**Conclusion:**

FAM, in axial or coronal planes with *R* = 2.0, is optimal for motion‐insensitive liver PDFF quantification.

## 1 INTRODUCTION

Noninvasive methods to quantify liver fat are urgently needed to address the growing public health challenge posed by metabolic dysfunction‐associated steatotic liver disease (MASLD). The prevalence of MASLD is rising rapidly, estimated at 32.4% worldwide^1^ and projected to reach 55.4% by 2040.^2^ Confounder‐corrected chemical shift‐encoded (CSE)–MRI methods to quantify liver proton‐density fat fraction (PDFF) are widely accepted as state of the art for accurate, precise, and noninvasive liver fat quantification.^3^, ^4^ An important byproduct of CSE–MRI methods is the simultaneous estimate of transverse magnetization decay rate, R2*.^5^ Confounder‐corrected R2* is widely accepted as an accurate and precise quantitative imaging biomarker of liver iron overload.^6^

Commercial confounder‐corrected CSE–MRI‐based PDFF and R2* quantification methods are widely available and have been extensively validated in phantom, animal, and human studies, including with comparison to biopsy.^3^, ^6^, ^7^ Most CSE–MRI methods are implemented as breath‐held 3D‐encoded Cartesian multi‐echo gradient echo acquisitions.^8^, ^9^ Unfortunately, many patients, particularly children or the infirm, are unable to hold their breath adequately, leading to severe motion artifacts. This limitation is significant because MASLD is the most common chronic liver disease in children in industrialized nations.^10^

A previously proposed CSE–MRI strategy that enables motion‐robust PDFF and R2* mapping is based on a 2D‐encoded Cartesian spoiled gradient echo acquisition, with sequential, short TR acquisitions.^11^ By using a short TR, each slice has a narrow temporal aperture that effectively freezes respiratory motion, even during free‐breathing. To avoid T1‐related bias in PDFF,^12^, ^13^ low flip angles were originally used with steady‐state 2D CSE–MRI.^11^ However, the combined effect of short TR, low flip angles, and intrinsically lower signal levels of 2D‐encoded acquisitions results in poor SNR performance.

To address this SNR limitation, Zhao et al.^14^ proposed a non–steady‐state, centric‐encoded flip angle modulation (FAM) strategy for 2D CSE–MRI that improves SNR by using the available magnetization more efficiently. FAM relies on an optimized flip angle schedule, applied across the phase‐encoding steps for each slice, to maximize SNR while also avoiding k‐space filtering effects and minimizing T1‐related bias. For clarity, we refer to conventional, breath‐held, 3D‐encoded CSE–MRI as *3D‐CSE* and the flip angle modulated, 2D‐encoded CSE–MRI method as *FAM*. Despite its early promise, the original FAM method did not include parallel imaging (PI) acceleration, limiting motion robustness. Further, FAM was only validated in the axial plane, which may lead to through‐plane motion and misregistration between slices in free‐breathing acquisitions.

For these reasons, there is a need to extend FAM by implementing it in combination with PI acceleration, with the goal to further shorten the temporal aperture of each slice, shorten overall scan time, and maximize motion robustness. Given the non–steady‐state approach in FAM, PI acceleration also requires fewer RF excitations, potentially enabling the use of higher overall flip angles. This allows for more efficient use of the available magnetization, mitigating SNR losses from PI acceleration. Further, it may be advantageous to use nonaxial imaging planes to reduce through‐plane respiratory motion.

Therefore, the purpose of this work is to implement, optimize and validate PI‐accelerated FAM for improved quantification of liver PDFF.

## 2 THEORY

The original FAM optimization scheme proposed by Zhao et al.^14^ creates a flip angle schedule with varying flip angles for each phase‐encoding step. This facilitates an optimized tradeoff between signal level, T1‐related bias, and filtering of k‐space in the phase‐encoding direction. More specifically, for a given flip angle schedule $\vec{\theta }$ with n excitations (i.e., n phase‐encoding TRs), the evolution of the fat and water magnetizations ${\vec{S}}_{\mathrm{fat}}$ and ${\vec{S}}_{\text{water}}$ can be simulated using the Bloch equations. For simplicity, perfect spoiling is assumed at the end of each TR, which is thought to be a reasonable assumption due to the combination of gradient spoiling and RF spoiling. For simulations, we used T1 values for liver fat and water previously published in literature (T1_water_ = 809 ms and T1_fat_ = 382 ms at 3.0 T).^15^ The flip angle schedule in FAM is then determined using the following optimization problem.^14^

$$\begin{array}{ll} {\vec{\theta }}_{\mathrm{opt}} & ={\text{argmin}}_{\vec{\theta }}\left(\lambda_{1}\sum_{i=1}^{n}{\left|S_{\mathrm{fat}}\left(\theta_{i}\right)-S_{\text{water}}\left(\theta_{i}\right)\right|}^{2}-\lambda_{2}\sum_{i=1}^{n}\left(S_{\mathrm{fat}}\left(\theta_{i}\right)\right.\right.\\ & \;+\left.{\left.S_{\text{water}}\left(\theta_{i}\right)\right)}^{2}+\lambda_{3}\sum_{i=1}^{n}\sum_{s=\mathrm{fat}}^{\text{water}}{\left(S_{s}\left(\theta_{i}\right)-f_{i}\cdot {\bar{S}}_{s}\right)}^{2}\right), \end{array}$$

where $\lambda_{1,2,3}$ are weighting coefficients for each term in the cost function, $\vec{f}$ is a desired k‐space profile, and ${\bar{S}}_{s}$ is a freely chosen scaling coefficient to best match a simulated signal profile ${\vec{S}}_{s}$ to $\vec{f}$. In this work, based on the analysis by Zhao et al.,^14^ we used the same weighting coefficients $\lambda_{1}=1000$, $\lambda_{2}=25$, and $\lambda_{3}=500$.

In this formulation, the first term penalizes T1‐related bias because differences in simulated fat and water signals at each excitation are due to the different T1 values for each species.^15^ The second term weights higher signal (and therefore, SNR). The third term is the most relevant for this work because it promotes adherence to the set k‐space profile $\vec{f}$. In the original work of Zhao et al.,^14^ the chosen profile $\vec{f}$ was Gaussian in both the k‐space and excitation domains because PI was not used and the excitations sampled k‐space uniformly. Here, we modify $\vec{f}$ to be Gaussian in k‐space only to accommodate nonuniform k‐space sampling. Further details and an illustrative example can be found in Figure S1.

With autocalibrated PI,^16^, ^17^ the center of k‐space is fully sampled, whereas lines in the outer regions of k‐space are undersampled by a nominal acceleration factor (*R*). The effective acceleration factor can be defined as the acquisition time that would be required if all of the k‐space was fully sampled, divided by the actual acquisition time. Conventionally, noise increases and SNR decreases in PI with the square root of the effective acceleration factor, in addition to any effects from coil sensitivity effects (g‐factor).^18^, ^19^ Therefore, PI does not typically improve SNR efficiency, which is defined as SNR divided by the square root of the acquisition time.^20^

**FIGURE 1:**
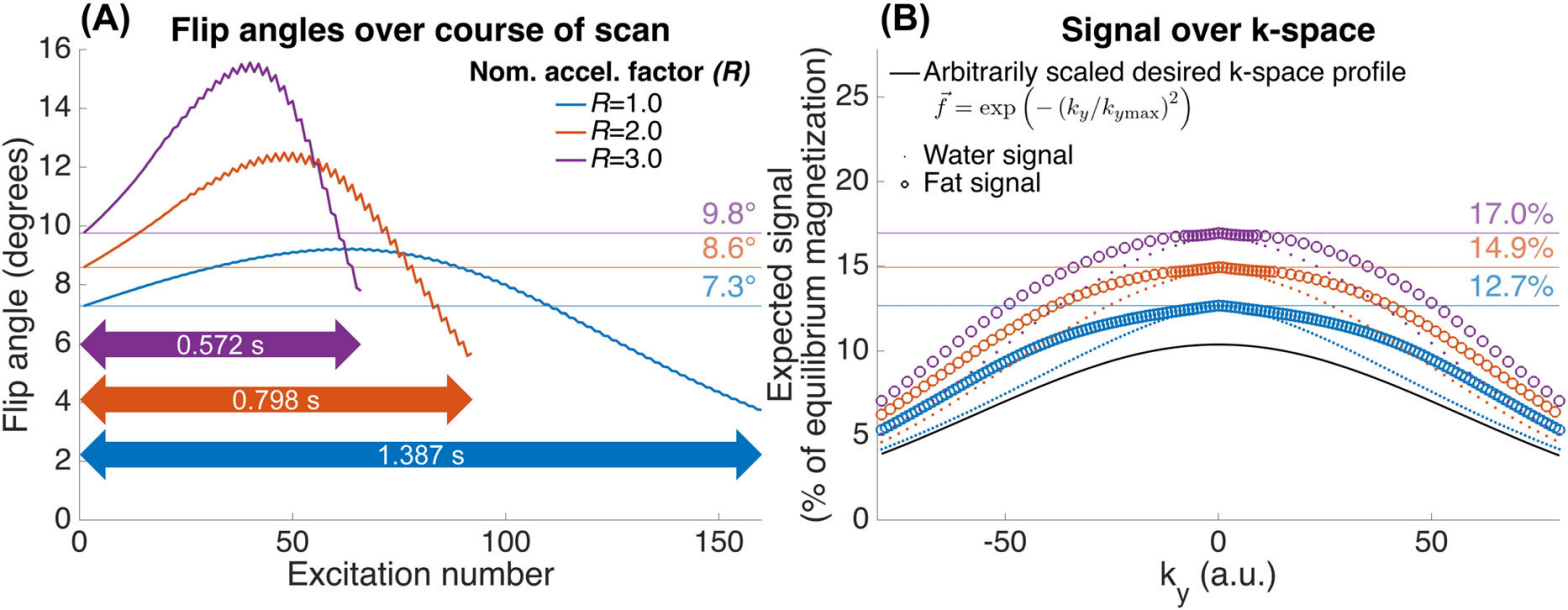
Flip angle schedules (subfigure A) and corresponding simulated k‐space profiles (subfigure B) for the sequences used in this study. Spacing of markers in subfigure B show how PI reduces acquisition of PE lines in the outer regions of k‐space, as well as the fully sampled autocalibration region in the center of k‐space. The acquisition of the autocalibration region results in effective acceleration factors lower than the nominal acceleration factors (*R*). FAM with higher accelerations can use higher flip angles (subfigure A) without accumulating significantly more T1 bias, because there are fewer excitations (PE lines). This translates into higher signal levels at the center of k‐space in our centric‐encoded acquisition (subfigure B) and offsets SNR loss due to parallel imaging. Higher accelerations also enable shorter temporal apertures. FAM, flip angle modulation; PE, phase encode; PI, parallel imaging.

However, in the context of in vivo free‐breathing imaging, PI acceleration also shortens the temporal aperture of each slice by acquiring fewer phase‐encoding lines, as illustrated in Figure 1A. This may improve image quality by reducing motion artifacts. Additionally, Bloch equation simulations show that T1‐related bias, or differences in ${\vec{S}}_{\mathrm{fat}}$ and ${\vec{S}}_{\text{water}}$, accumulate over multiple excitations during this non–steady‐state acquisition because the fat and water magnetizations both start at equilibrium. Because fewer excitations are required when using PI, larger flip angles may be used without accumulating additional T1‐related bias, with a resulting increase in SNR performance. This is illustrated in Figure 1, which shows that the flip angles of the optimized flip angle schedule are generally higher for FAM with higher *R* (Figure 1A). The use of increased flip angles leads to higher signal levels at the center of k‐space, without significantly increased differences between ${\vec{S}}_{\mathrm{fat}}$ and ${\vec{S}}_{\text{water}}$ (Figure 1B). Importantly, in contrast to conventional PI‐accelerated methods, the use of higher flip angles in accelerated FAM may help offset, at least in part, the sqrt(effective acceleration) decrease in SNR typically associated with PI. This may then improve SNR efficiency. Specifically, we expect the SNR decrease to be approximately offset by a factor of the sine of the initial flip angle because this determines the signal level at the center of k‐space in our non–steady‐state centric‐encoded acquisition (see Figure 1A,B).

One potential challenge affecting FAM may be B1+ inhomogeneity in the liver. Previous work^21^ has measured actual flip angles to be 59% to 113% of prescribed flip angles (fifth to 95th percentile of observations at 3 T). However, Bloch equation simulations (see Figure S2) show that even at these extreme ranges of B1+ inhomogeneity, the signal profiles produced by optimized FAM flip angles show reasonable filtering effects. Further, even with any amount of B1+ inhomogeneity, the non–steady‐state centric encoding of FAM maintains minimal T1‐related bias.

## 3 METHODS

We performed phantom studies and prospective in vivo studies to evaluate the bias, repeatability, and noise performance of FAM.

For both phantom and in vivo studies, we acquired FAM images in the axial, coronal, and sagittal planes, without any acceleration, that is, *R* = 1.0, similar to Zhao's original unaccelerated implementation,^14^ as well as with PI acceleration with *R* = 2.0 and 3.0. A previously validated, commercially available 3D‐CSE method^22^, ^23^, ^24^ (IDEAL IQ, GE HealthCare, Waukesha, WI) was also acquired in the axial plane as a reference. Key imaging parameters are given in Table 1. Flip angles were re‐optimized for each configuration of imaging parameters by minimizing the generalized FAM cost function above. Acquisitions were obtained using a high‐performance whole‐body 3.0 T system (Signa Premier, GE HealthCare). Some of the methods and results below were previously reported in scientific abstracts.^25^, ^26^

**TABLE 1:** Imaging parameters for the acquisitions used in this study.

Acquisition	BH 3D‐CSE	FB 2D FAM Axial	FB 2D FAM Coronal	FB 2D FAM Sagittal
TE (ms)	TE_1_ = 0.97, ∆TE = 0.78, *N*_TE_ = 6	TE_1_ = 0.98, ∆TE = 1.2, *N*_TE_ = 6	TE_1_ = 1.1, ∆TE = 1.2, *N*_TE_ = 6	TE_1_ = 1.1, ∆TE = 1.0, *N*_TE_ = 6
TR (ms)	6.28	8.67	8.70	7.69
Total bandwidth (kHz)	250	250	250	250
Flip angle (degrees)	3	Variable (see Figure 1A)		
FOV (cm × cm)	40 × 32	40 × 40	40 × 40	32 × 32
Slice thickness (mm)	8.0	8.0	5.0	4.0
Acquisition matrix size	192 × 128	160 × 160	160 × 160 (no phase wrap–factor = 1.2)	92 × 92
Nominal acceleration factor (R)	2.0 in PE direction and 2.0 in slice direction	1.0, 2.0, 3.0 in PE direction only		
Effective acceleration factors (acquisition time for full sampling/actual acquisition time)	2.8	1.0, 1.7 (24 ACS lines), 2.4 (18 ACS lines)	1.0, 1.8 (24 ACS lines), 2.5 (18 ACS lines)	1.0, 1.6 (24 ACS lines), 2.1 (18 ACS lines)
Temporal aperture per slice (s) (*R* = 1.0, 2.0, 3.0 for FAM sequences)	18.50	1.387, 0.798, 0.572	1.688, 0.948, 0.670	0.708, 0.446, 0.330
Typical number of slices	32	32	60	80
Total acquisition time (s) (*R* = 1.0, 2.0, 3.0 for FAM sequences)	18.50	44.4, 25.5, 18.3	101.3, 56.9, 40.2	56.6, 35.7, 26.4

Abbreviations: ACS, autocalibration signal; BH, breath‐held; CSE, chemical shift‐encoded; FAM, flip angle modulated; FB, free‐breathing; PE, phase encode.

### 3.1 Phantom study

The purpose of phantom experiments was to evaluate the bias and noise performance of PDFF quantification, specifically addressing two technical challenges, that is, T1‐related bias and PDFF quantification with high R2*. T1 bias is a well‐known confounder in fat quantification^12^ and is considered in the cost function in FAM's numerical optimization. Further, due to the non–steady‐state acquisition used in FAM, all echoes must be acquired in a single echo train, unlike for 3D‐CSE acquisitions that may use multiple echo trains, especially at 3.0 T. The requirement to use a single echo train leads to relatively long TE spacing, which may decrease the performance of FAM, especially in the presence of high R2*. For these reasons, phantom experiments were conducted with two different phantoms: a combined PDFF‐T1 phantom, and a combined PDFF‐R2* phantom, as described below.

#### 3.1.1 Description of phantoms

A commercially available combined PDFF‐T1 phantom (available by special order, Calimetrix, Madison, WI), containing a 2D grid of 16 cylindrical vials of gels with simultaneously controlled combinations of PDFF (0%, 10%, 20%, 30%) and T1_w_ (T1_water_) (200, 600, 1000, 1400 ms), was used. The PDFF values in the phantom cover a wide range of liver PDFF values observed in vivo. The T1_w_ values cover a wide range of T1_w_ observed in healthy and diseased liver, including pre‐ and post‐contrast.^27^, ^28^ This allowed us to evaluate T1‐related bias in a physiologically relevant range.

We also used a separate commercially available PDFF‐R2* phantom (model 725, Calimetrix, Madison, WI) containing 16 cylindrical vials of gels with simultaneously controlled combinations of PDFF (0%, 10%, 20%, 30%) and R2* (50, 150, 350, and 600 s^−1^). This allowed us to evaluate performance over a wide range of R2* values observed in the presence of liver iron overload, over a relevant range of clinically observed fat concentrations.

#### 3.1.2 Phantom experiments

As described above, for each phantom, FAM images were acquired in three imaging planes, each at increasing PI acceleration (*R* = 1.0, 2.0, and 3.0) for a total of nine acquisitions. A 48‐channel head coil (GE HealthCare) was used. Each phantom was rotated inside the coil as imaging planes changed to acquire slices orthogonal to the long axis of the vials. We also imaged the phantoms using 3D‐CSE in the axial plane. To assess noise performance as PI acceleration increased, each FAM acquisition was repeated 10 times without repositioning, for a total of 90 FAM acquisitions for each phantom.

An offline graph‐cut algorithm^29^ was used to reconstruct PDFF and R2* maps. Hybrid fitting (i.e., a weighted average of magnitude and complex fitting^23^) was used. To account for the effect of temperature on the fat spectrum peaks, a shift of 0.11 ppm in the fat spectrum^30^ was applied, assuming a temperature of 20°C. Other than this shift, the offline algorithm is functionally equivalent to the offline and vendor‐provided online algorithms used in vivo (see below), which are calibrated for fat peaks at typical body temperature. Voxel‐wise standard deviations (SDs) across the 10 repeated acquisitions were computed from the PDFF maps as a surrogate for noise.

Circular regions of interest (ROIs) of 12.5 mm diameter were drawn on the resulting PDFF, R2*, and voxel‐wise PDFF SD maps at the center of each vial of the phantom. Summary values for each ROI were computed by averaging voxels within the ROI on each quantitative map. Bias was evaluated by comparing summary PDFF and R2* values to the vials' nominal PDFF and R2* values. Noise performance with increasing PI acceleration was evaluated by comparing summary values for voxel‐wise PDFF SDs.

### 3.2 In vivo study

The goal of our in vivo study was to demonstrate the feasibility of free‐breathing FAM through evaluation of image quality, bias, test–retest repeatability, and noise performance of PDFF quantification. To achieve this goal, we conducted a prospective in vivo study after obtaining institutional review board approval and informed written consent.

Because the primary goal was to demonstrate the feasibility of the PI‐accelerated FAM technique for PDFF mapping, a wide range of liver PDFF was desired in volunteers. To achieve this, we recruited volunteers with a wide range of body mass index (BMI), given the known correlation of BMI with liver fat content.^31^, ^32^, ^33^ Specifically, we recruited volunteers with BMI in the following ranges: <20, 20 to 25, 25 to 30, 30 to 35, and > 35 kg/m^2^, in equal proportions from a local institutional review board–approved volunteer database.

Breath‐held 3D‐CSE was acquired as the reference for PDFF in all volunteers. Free‐breathing FAM acquisitions were performed as above in all three orthogonal planes, each with *R* = 1.0, 2.0, or 3.0, for a total of nine FAM acquisitions. A 30‐channel flexible coil (GE HealthCare) was used, in addition to the spine coil built into the system couch. To evaluate test–retest repeatability, all 3D‐CSE and FAM acquisitions were repeated after removing subjects from the bore, repositioning the subjects, and repeating localizers. In vivo PDFF and R2* maps were reconstructed using the vendor‐provided online algorithm,^22^, ^23^, ^24^ maximizing the clinical relevance of this work. Within an exam, no acquisitions were repeated due to motion artifacts to avoid biasing ratings in the reader study (see below).

#### 3.2.1 Reader study

All vendor‐reconstructed PDFF and R2* maps without significant fat–water swaps from the test acquisitions (i.e., before patient repositioning) were evaluated by three radiologists (J.S., J.F.H., L.M.) with 5 to 12 years of experience in abdominal MRI. Ratings were fully randomized and blinded to the acquisition techniques (e.g., acceleration factor, 3D‐CSE vs. FAM). A 5‐point Likert scale was used to evaluate overall image quality, qualitative SNR, and motion artifacts (5 = excellent quality, no noise/artifacts; 4 = very good quality, minor noise/artifacts; 3 = moderate quality, moderate noise/artifacts; 2 = fair but still diagnostic quality, major noise/artifacts; 1 = nondiagnostic quality, severe noise/artifacts). Inter‐reader reliability between the three readers was assessed using intraclass correlation coefficient with a two‐way random effects model, including averaged measures and absolute agreement (ICC(2,k)). For paired comparisons between CSE techniques, ratings were pooled between all readers (not averaged) and two‐tailed Wilcoxon signed‐rank tests were calculated. Statistical calculations were performed with Pingouin version 0.5.5.^34^ The threshold for statistical significance was set at *p* = 0.05.

#### 3.2.2 Quantitative analysis

For quantitative analysis, when vendor‐provided online reconstructions showed significant fat–water swaps, reconstructions were re‐processed offline with a confounder‐corrected graph‐cut algorithm.^29^ This is the same algorithm as was used for phantoms but without a temperature‐related shift for the fat spectrum peaks because that is not necessary in vivo. For each acquisition, two analysts with 1 to 2 years of experience in the analysis of quantitative abdominal MRI (R.V.S., a physician; A.F., a medical student) placed one ROI within each of the nine Couinaud liver segments^35^ on all reconstructed PDFF and R2* maps. All analyses were conducted with medical image viewer software (Horos, Annapolis, MD) under the supervision of a board‐certified radiologist (J.S.) with 12 years of experience in abdominal MRI. ROIs were elliptical, approximately 2.3 to 4.3 cm^2^ in area, and avoided bile ducts, large blood vessels, and obvious artifacts. ROIs were propagated between the PDFF and R2* maps using copy‐and‐paste functionality to ensure perfect colocalization. As done in the phantom, summary quantification values for each ROI were computed by averaging PDFF and R2* values within ROIs.

The quantitative interchangeability of the offline and vendor‐provided online algorithms was verified by paired comparison of summary PDFF values. Two axial, two coronal, and two sagittal acquisitions without fat–water swaps in the online reconstruction were randomly selected. From these six acquisitions, a total of 54 summary PDFF values were compared between online and offline reconstructions by linear fitting.

The bias of PDFF and R2* measurements was evaluated by comparing summary values for the same liver segments between the 3D‐CSE and FAM acquisitions in Bland–Altman analysis. The 95% limits of agreement (LoA) were calculated as 1.96 times the SD of all summary value differences between 3D‐CSE and FAM.

To evaluate repeatability, summary PDFF and R2* values were compared between test and retest acquisitions using Bland–Altman analysis. The repeatability coefficient (RC) was calculated as 1.96 times the SD of all test–retest summary value differences. Significance of differences in RC were tested by comparing the variances of test–retest differences with a two‐tailed Levene's test, with significance level set at *p* = 0.05.

Finally, intervoxel SDs (i.e., SD among the voxels within an ROI) for PDFF maps were measured as a surrogate for noise, under the assumption that true PDFF heterogeneity should be minimal within our small ROIs. Note that this is different from the noise measurements made in phantoms, where due to the absence of motion, SDs could be calculated voxel‐wise across repeated acquisitions.

## 4 RESULTS

### 4.1 Phantom study

**FIGURE 2:**
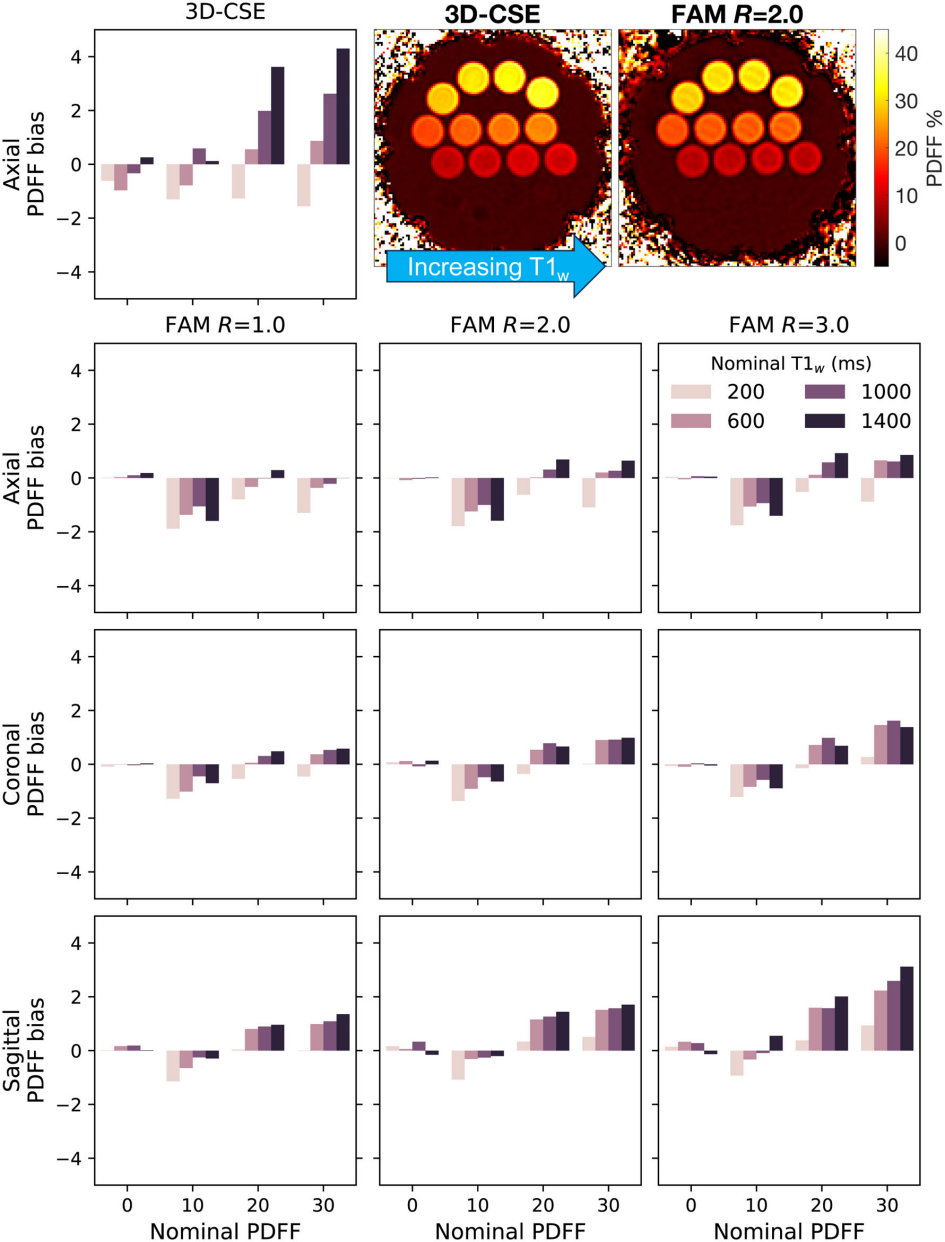
In a PDFF‐T1 phantom, FAM shows reduced T1 bias in PDFF quantification compared to 3D‐CSE. A phantom modulated in PDFF (0% to 30%) and T1_w_ (200 to 1400 ms) was imaged in 3D‐CSE and FAM, and ROIs were drawn on the vials of the phantom and compared to the nominal PDFF of each vial to determine bias. 3D‐CSE shows moderate T1 bias, ranging in bias from approximately −1% to 4% as T1_w_ increases, whereas biases in FAM increase only approximately 1% to 2% over the entire T1_w_ range. CSE, chemical shift‐encoded; PDFF, proton‐density fat fraction; T1_w_, T1_water_.

Results from phantom experiments generally showed low bias relative to nominal PDFF and R2* values. In the PDFF‐T1 phantom, FAM showed reduced T1‐related PDFF quantification bias compared to 3D‐CSE (Figure 2). In the PDFF‐R2* phantom, FAM showed low bias at R2* values up to 150 s^−1^; however, at values of R2* at 350 s^−1^ and particularly at 600 s^−1^, FAM showed artifacts and bias in PDFF quantification (Figure S3) and R2* quantification (Figure S4). Note all phantom results are from the temperature‐corrected offline fat–water reconstruction; using non–temperature‐corrected online reconstruction underestimates PDFF by approximately 2.5% in the 30% nominal PDFF vials compared to the corrected offline method.

**FIGURE 3:**
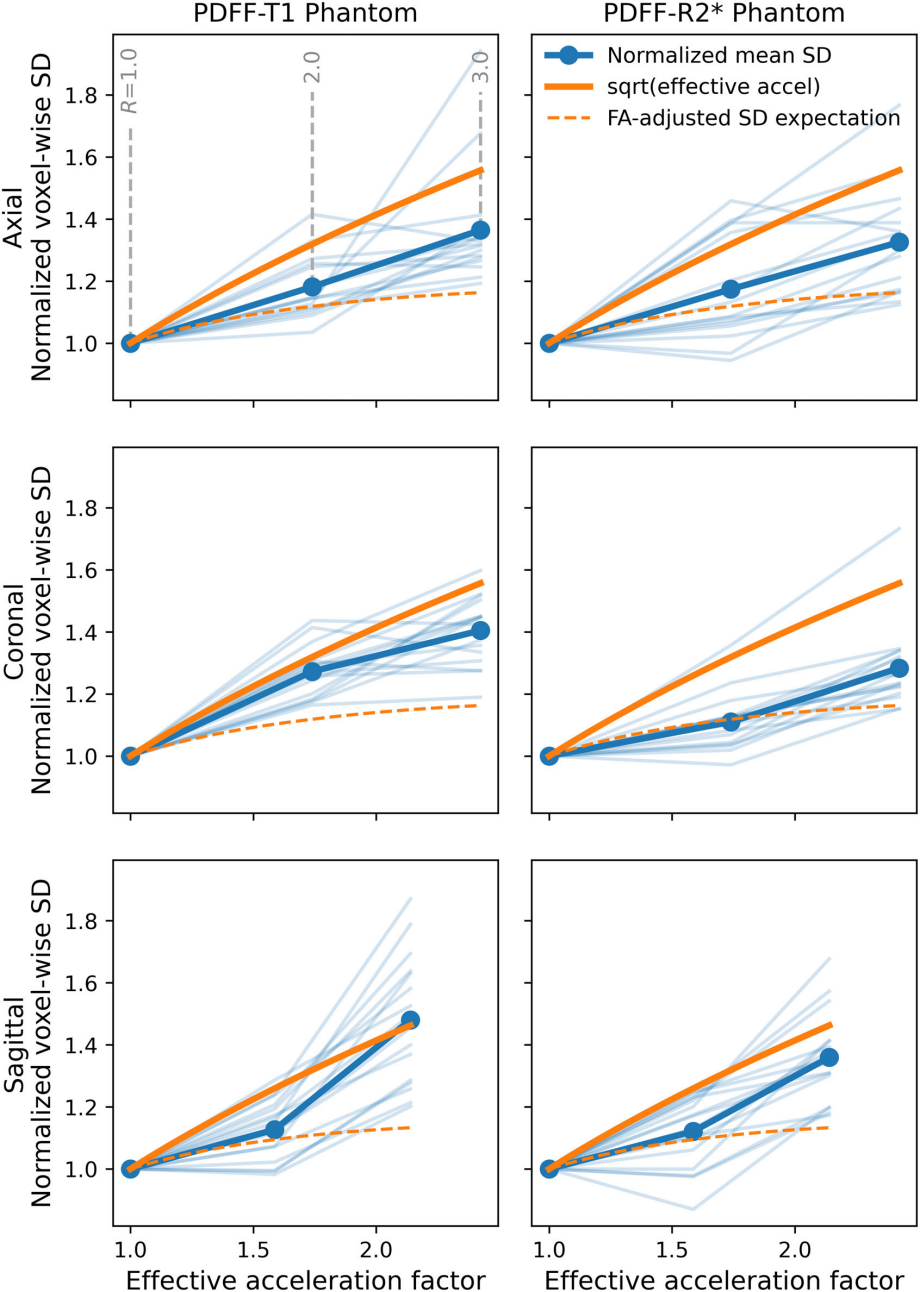
FAM with acceleration shows lower noise increases than the conventional sqrt(effective acceleration) relation in PI. Ten acquisitions of the PDFF‐T1 and PDFF‐R2* phantom were obtained with FAM at *R* = 1.0, 2.0, and 3.0 without repositioning, and voxel‐wise SDs on the resultant PDFF maps calculated across the repetitions as a surrogate for noise. ROIs were then drawn on each vial of each phantom and summary SD values were obtained by averaging across each ROI. The summary SDs were normalized to *R* = 1.0 to evaluate the increase in noise as *R* increases. Each thin, light blue line represents an individual vial ROI, and the thick blue line represents the average across the vials. The conventional sqrt(effective acceleration) relation is plotted in the thick orange line, and the dashed orange line adjusts this relation for the increased FAs used at higher acceleration factors (see Figure 1 and Theory section). Overall, accelerated FAM achieves lower noise than expected with PI, and generally approaches the theoretical performance that considers the increased FAs. *R* = 2.0 acquisitions appear to especially demonstrate strong noise performance. FA, flip angle; ROI, region of interest; SD, standard deviation.

As shown in Figure 3, voxel‐wise PDFF SDs increased more slowly than the conventional sqrt(effective acceleration) relation typical to parallel imaging.^18^, ^19^ Acquisitions with nominal acceleration *R* = 2.0 (effective acceleration of 1.6 to 1.7) had lower noise than conventionally expected for this effective acceleration, and approached the theoretical noise performance that considers the increased flip angles used in FAM with PI (see Figure 1 and Theory section).

### 4.2 In vivo study

**FIGURE 4:**
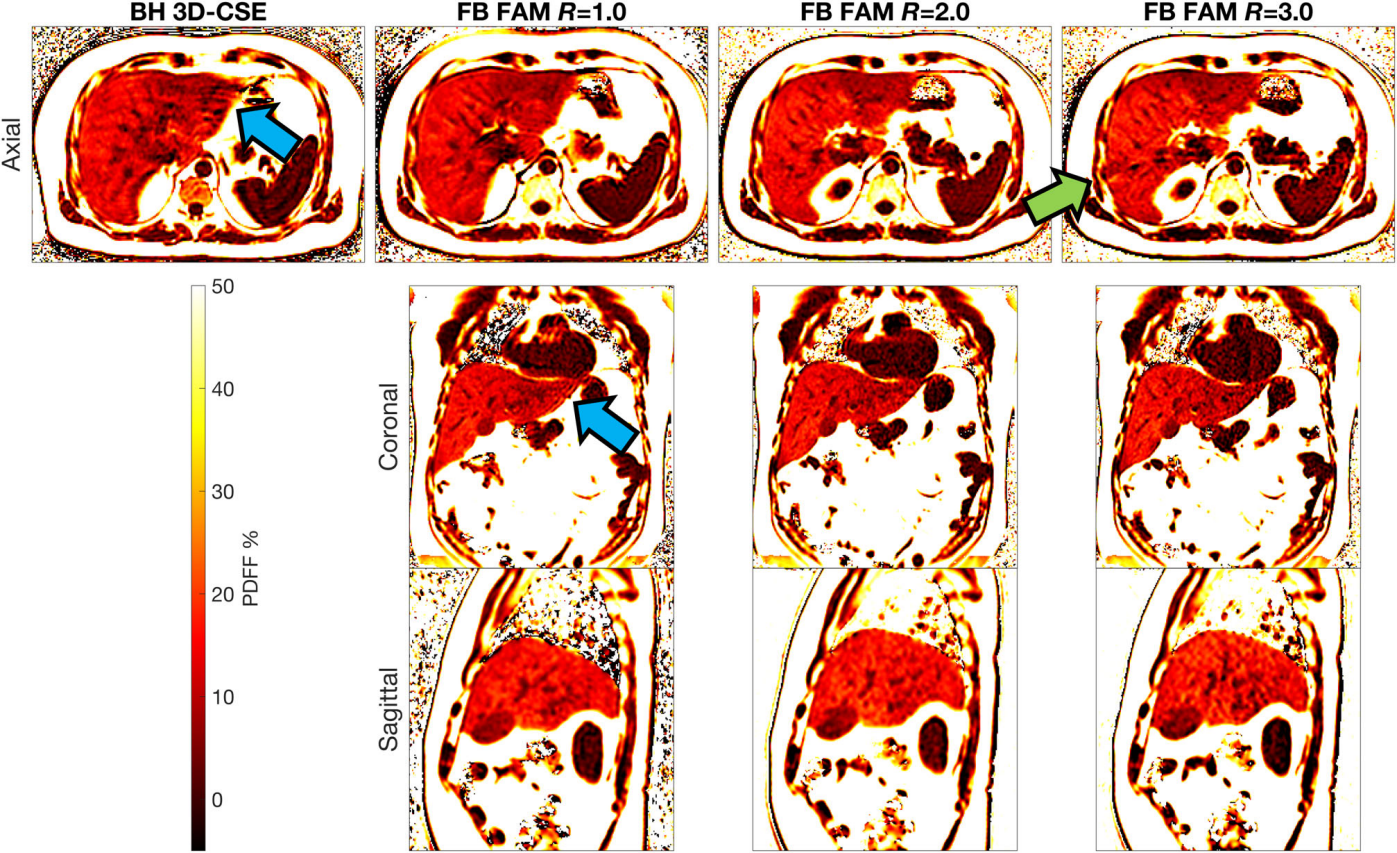
Representative in vivo PDFF maps for the BH 3D‐CSE and FB FAM acquisitions in this study. FAM was acquired in three imaging planes and at three values of *R*. FAM images generally show good image quality and motion robustness, whereas 3D‐CSE images are also generally high quality in subjects who can sustain a breath hold. 3D‐CSE, and sometimes FAM at *R* = 1.0, show cardiac motion artifacts in the left liver lobe (blue arrows). FAM at *R* = 3.0 sometimes shows residual aliasing (green arrow). BH, breath‐held; FB, free‐breathing; *R*, nominal acceleration factors.

Ten volunteers (age range 28 to 63 years, 50% male/50% female, BMI 19.3 to 41.7 kg/m^2^, with two volunteers each in the BMI groups <20, 20 to 25, 25 to 30, 30 to 35, and > 35 kg/m^2^) were successful recruited. Representative images of the PDFF maps obtained in this study are shown in Figure 4.

**FIGURE 5:**
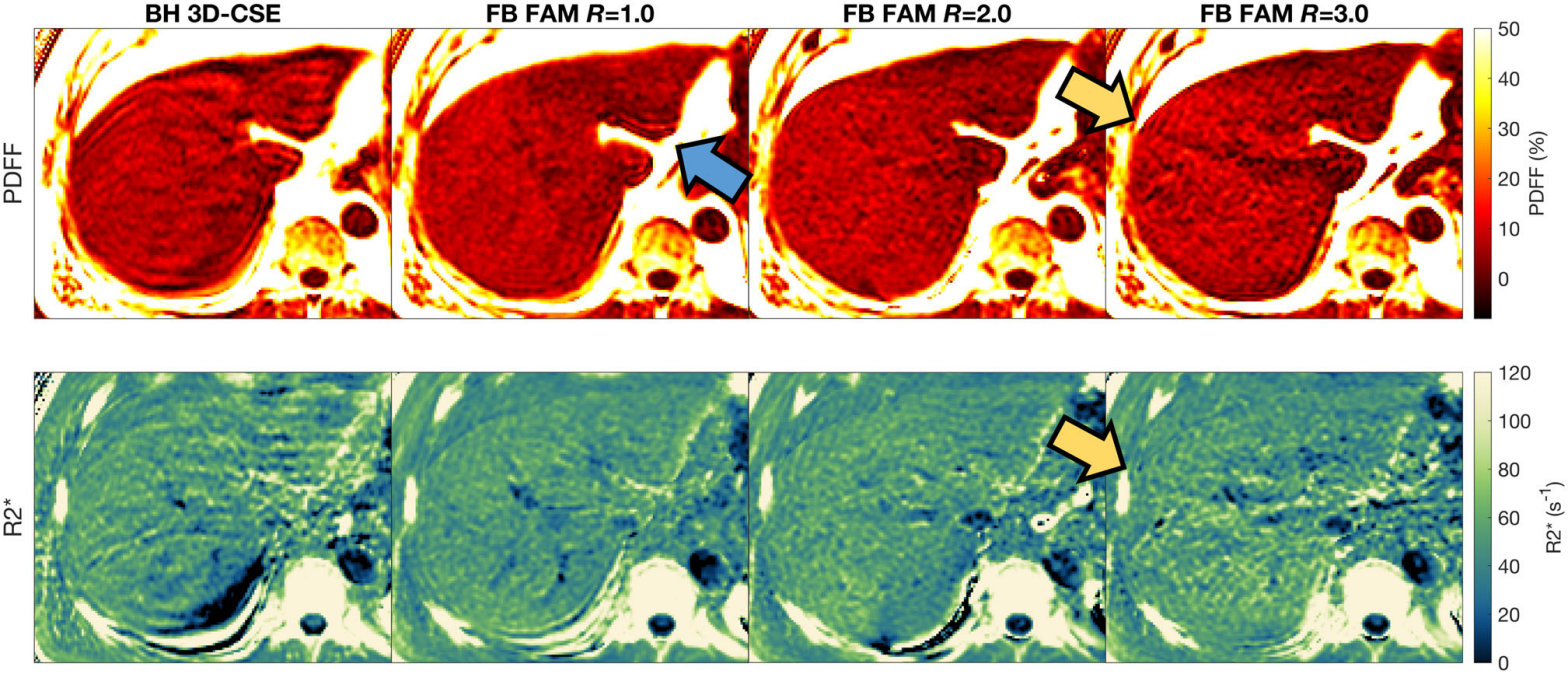
Representative axial images showing various artifacts encountered in vivo in this study. BH 3D‐CSE shows the most pronounced artifacts throughout the liver in PDFF and R2* maps, possibly due to poor breath holding and cardiac motion. FB FAM is essentially free of respiratory motion artifacts at all values of *R*; FAM with *R* = 1.0 shows some less severe, localized cardiac motion artifacts in the PDFF map (blue arrow). FAM with *R* = 2.0 may provide a good compromise between motion‐robustness, SNR, and overall image quality (see also reader study). FAM with *R* = 3.0 shows residual aliasing in the PDFF and R2* maps (yellow arrows).

With respect to image quality, 3D‐CSE exhibited frequent artifacts throughout the liver, despite attempted breath holds by the volunteers, as illustrated in Figure 5. FAM showed consistently good image quality, with minimal motion‐induced artifacts such as ghosting, even during free‐breathing. Figure 5 also illustrates that unaccelerated FAM sometimes showed some localized motion artifacts in the left liver lobe near the heart, which was generally not present in FAM with higher *R* (2.0 and 3.0). Residual aliasing from high acceleration was observed in some *R* = 3.0 images. These observations on image quality and artifacts are more systematically examined in the reader study.

#### 4.2.1 Reader study

**FIGURE 6:**
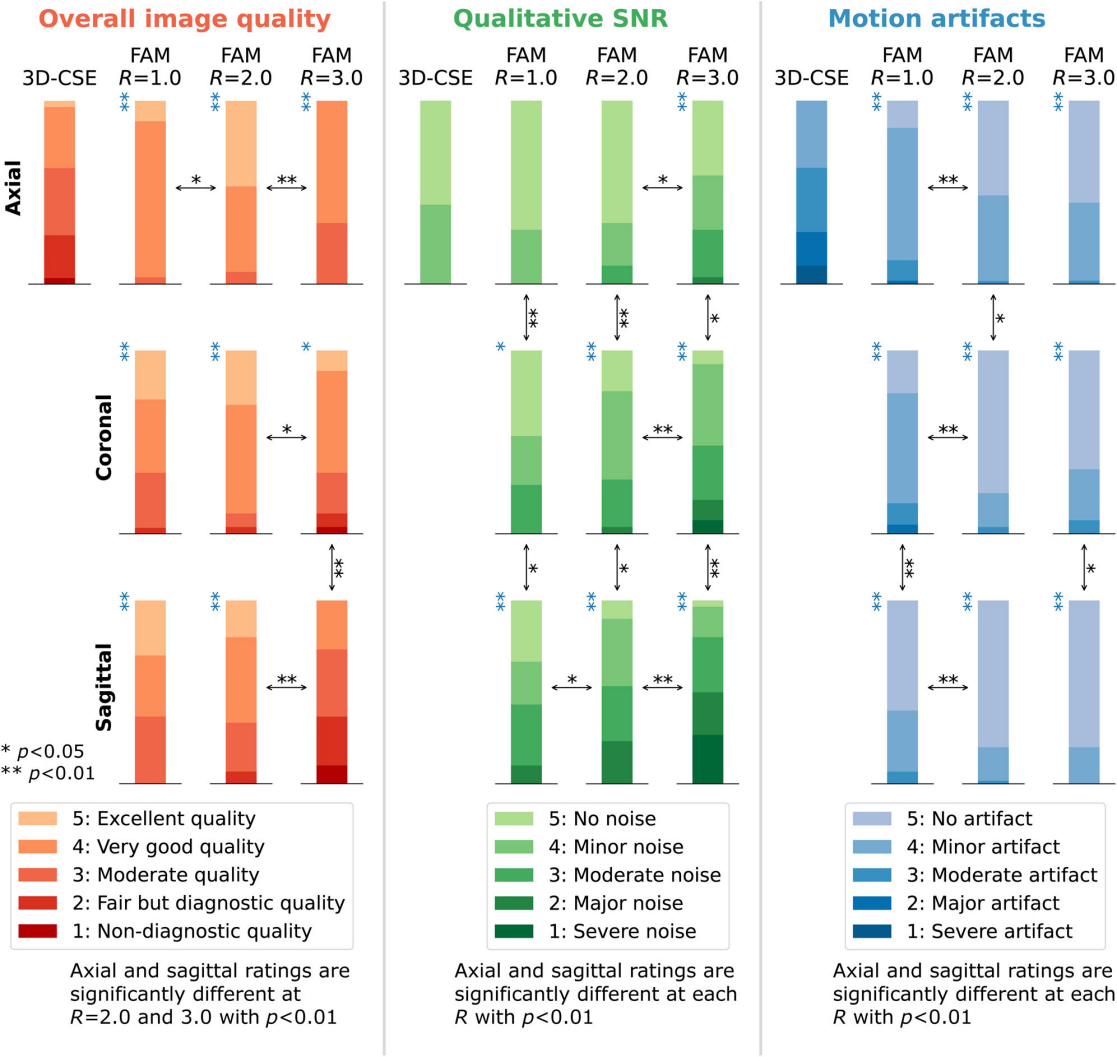
Reader study results show the strong performance of *R* = 2.0 PDFF maps in overall image quality, qualitative SNR, and lack of motion artifacts. Stacked bar plots show the distribution of Likert scale ratings on a subset of PDFF maps acquired in this study, pooled from three radiologist readers. Two‐tailed Wilcoxon signed‐rank tests were used in paired comparisons between CSE methods. Blue asterisks to the top left of each stacked bar show significance of comparison with BH 3D‐CSE. In overall image quality, all FB FAM methods other than sagittal with *R* = 3.0 show statistically significant improvement versus BH 3D‐CSE. In qualitative SNR, axial FAM with *R* = 1.0 and 2.0 had similar SNR as 3D‐CSE, whereas other FAM methods were worse. In the axial and coronal planes, qualitative SNR did not significantly worsen moving from *R* = 1.0 to 2.0 but did significantly worsen in all imaging planes from *R* = 2.0 to 3.0. In motion artifacts, all FAM methods are significantly better than 3D‐CSE. In each imaging plane, moving from *R* = 1.0 to 2.0 significantly reduced artifacts but not when moving from *R* = 2.0 to 3.0.

Readers evaluated 96/100 of the test acquisitions, excluding four image series due to extensive fat–water swaps. Figure 6 shows reader study results for PDFF maps. For overall image quality, all FAM techniques except sagittal with *R* = 3.0 showed improved image quality compared to 3D‐CSE. In the axial plane, FAM with *R* = 2.0 showed improved image quality compared to both *R* = 1.0 (*p* < 0.05) and 3.0 (*p* < 0.01). In the coronal and sagittal planes, both *R* = 1.0 and 2.0 acquisitions showed comparable image quality, which was superior to *R* = 3.0 (*p* < 0.05 for coronal and *p* < 0.01 for sagittal). Altogether, FAM with *R* = 2.0 in the axial and coronal planes received the best ratings for overall image quality.

For qualitative SNR of PDFF maps, 3D‐CSE and axial FAM with *R* = 1.0 and 2.0 had the best SNR ratings, with no statistically significant differences between the ratings received. Qualitative SNR is lower for coronal acquisitions versus axial (*p* < 0.05), and sagittal acquisitions are worse than coronal (*p* < 0.05). In axial and coronal acquisitions, there is no statistically significant difference in ratings between *R* = 1.0 and 2.0 images, whereas in all imaging planes, *R* = 3.0 images are worse (*p* < 0.05, 0.01, 0.01 for axial, coronal, and sagittal planes, respectively).

For motion artifacts, all FAM techniques showed significantly reduced motion artifacts compared with 3D‐CSE. In each imaging plane, FAM with *R* = 2.0 showed significantly fewer motion artifacts than FAM with *R* = 1.0 (*p* < 0.01), but differences between *R* = 2.0 and 3.0 were not significant.

Similar (but not identical) trends between imaging planes and acceleration factors were also found for R2* maps in overall image quality, qualitative SNR, and motion artifact ratings, as seen in Figure S5.

Agreement between readers was good, with ICC(2,k) of 0.76 to 0.84 for the three rating criteria used.

#### 4.2.2 Quantitative analysis

Of the 180 FAM datasets acquired, nine datasets (5%) showed pronounced fat–water swaps in the online reconstruction impacting evaluation of the liver, whereas no 3D‐CSE datasets showed extensive fat–water swaps. FAM datasets with pronounced swaps were successfully re‐processed with an offline fitting algorithm for quantitative analysis, as described above. A linear fit between 54 summary PDFF values from the two reconstruction methods, where neither method showed fat–water swaps, demonstrated tight agreement, with a slope with 95% confidence bounds (0.99, 1.01), intercept with bounds (−0.12, 0.07), and Pearson correlation coefficient (R^2^) of 0.999. Note that this comparison is between online fat–water fitting and offline fitting without temperature‐related shift for fat spectrum peaks, which is appropriate in vivo.

**FIGURE 7:**
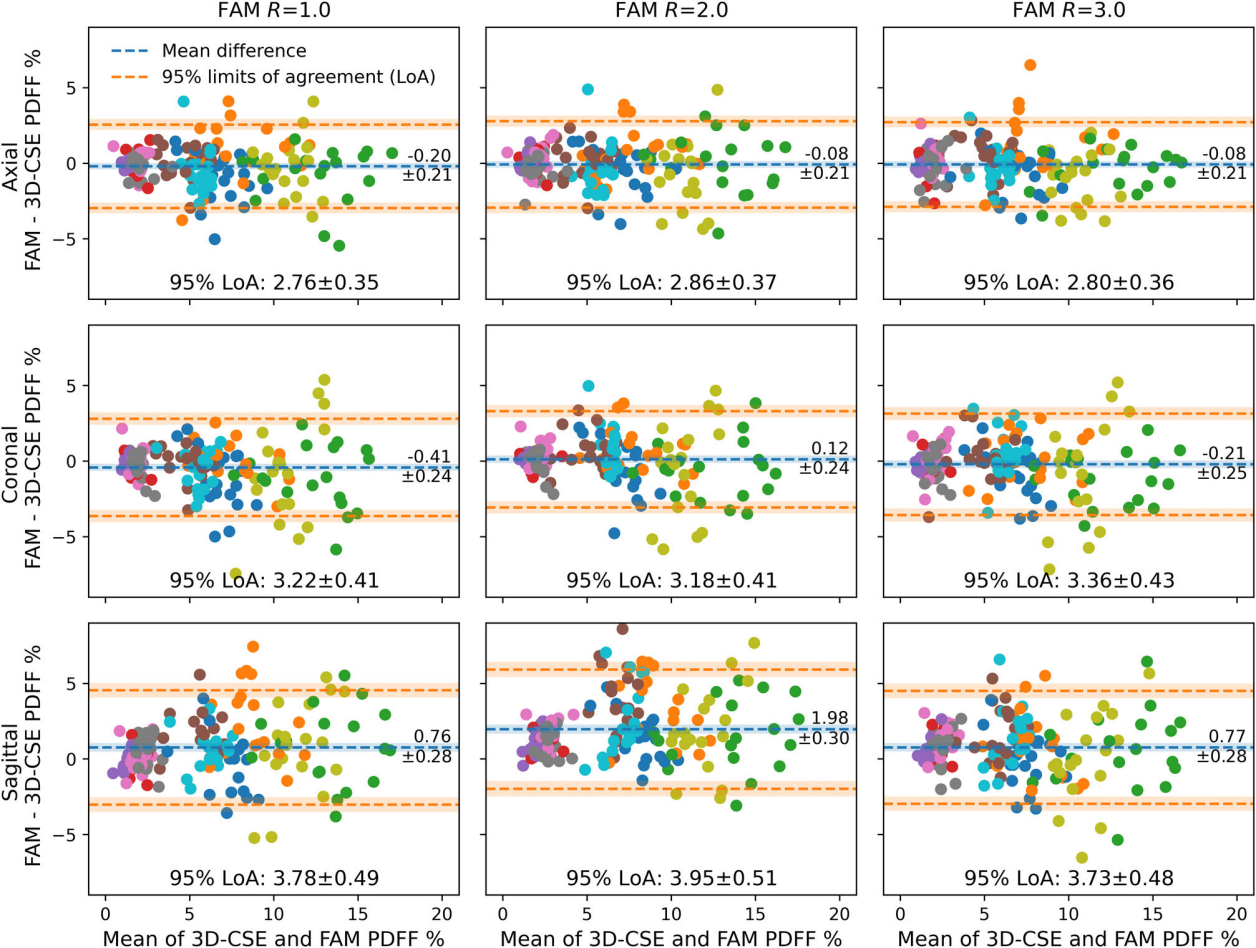
3D‐CSE and FAM show strong agreement in vivo for liver PDFFs. The figure shows Bland–Altman analysis of PDFF summary ROI values for various free‐breathing FAM acquisitions compared to the reference, 3D‐CSE acquired in a breath hold. Mean differences, 95% limits of agreement, and corresponding 95% confidence intervals are given in the plot for each method. FAM shows generally good agreement with the 3D‐CSE reference at all accelerations and orientations. The 95% LoA between FAM methods and 3D‐CSE is close to the test–retest repeatability of the 3D‐CSE method itself, as seen in this study and previously reported in literature. Different point colors indicate different imaged subjects. LoA, limits of agreement.

Figure 7 summarizes the comparison of FAM and 3D‐CSE to assess for bias, demonstrating that FAM acquisitions had good agreement with 3D‐CSE for PDFF quantification in all imaging planes (mean bias between −0.4% and 2.0% PDFF; 95% LoA between 2.8 and 4.0% PDFF). Figure S6 shows that this was especially true in the right lobe of the liver (mean bias between −0.1% and 1.7% PDFF; 95% LoA between 1.7% and 3.0% PDFF). Although the subjects recruited for this study did not represent a wide range of R2* (30 to 80 s^−1^), Figure S7 illustrates that FAM showed generally good agreement with 3D‐CSE in R2* quantification in this limited range (mean bias between −4.2 and 1.6 s^−1^; 95% LoA between 10.5 and 15.1 s^−1^).

**FIGURE 8:**
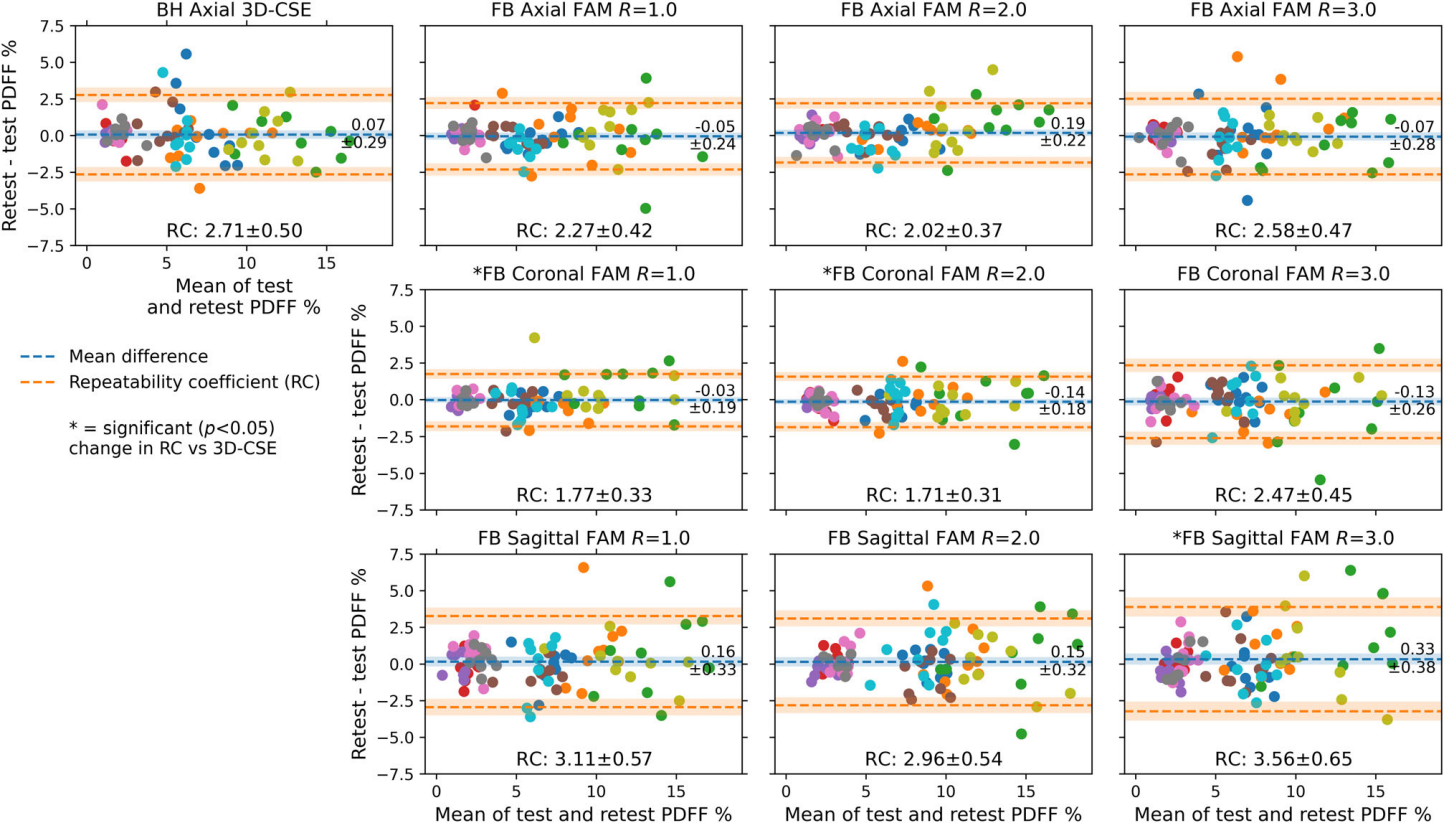
FB FAM generally shows similar or improved test–retest repeatability in PDFF quantification compared to BH 3D‐CSE. The figure shows Bland–Altman test–retest repeatability analysis for PDFF summary values from the reference 3D‐CSE and various FAM methods. Mean differences, repeatability coefficients, and corresponding 95% confidence intervals are given in the plot for each method. Coronal FAM at *R* = 1.0 and 2.0 show statistically significant improvement in repeatability compared to 3D‐CSE, whereas sagittal FAM at *R* = 3.0 is significantly worse. Other FAM methods show similar repeatability as 3D‐CSE. Acquisitions with *R* = 2.0 show similar repeatability as *R* = 1.0 acquisitions, while enabling shorter scans and a shorter per‐slice temporal aperture. Different point colors indicate different imaged subjects.

Figure 8 summarizes test–retest repeatability results, demonstrating that free‐breathing FAM‐based PDFF quantification was significantly superior in test–retest repeatability than 3D‐CSE for coronal FAM with *R* = 1.0 and 2.0, and significantly worse than 3D‐CSE in sagittal FAM with *R* = 3.0 (all with *p* < 0.05). For all other FAM configurations, repeatability was not significantly different from 3D‐CSE. FAM generally shows similar test–retest repeatability as 3D‐CSE for R2* quantification (Figure S8).

As shown in Figure S9, 3D‐CSE showed significant degradation (*p* < 0.01) in PDFF repeatability in the left lobe of the liver, compared to the right lobe. All FAM methods, other than sagittal with *R* = 3.0, did not show significant degradation in repeatability between the left and right liver lobes.

**FIGURE 9:**
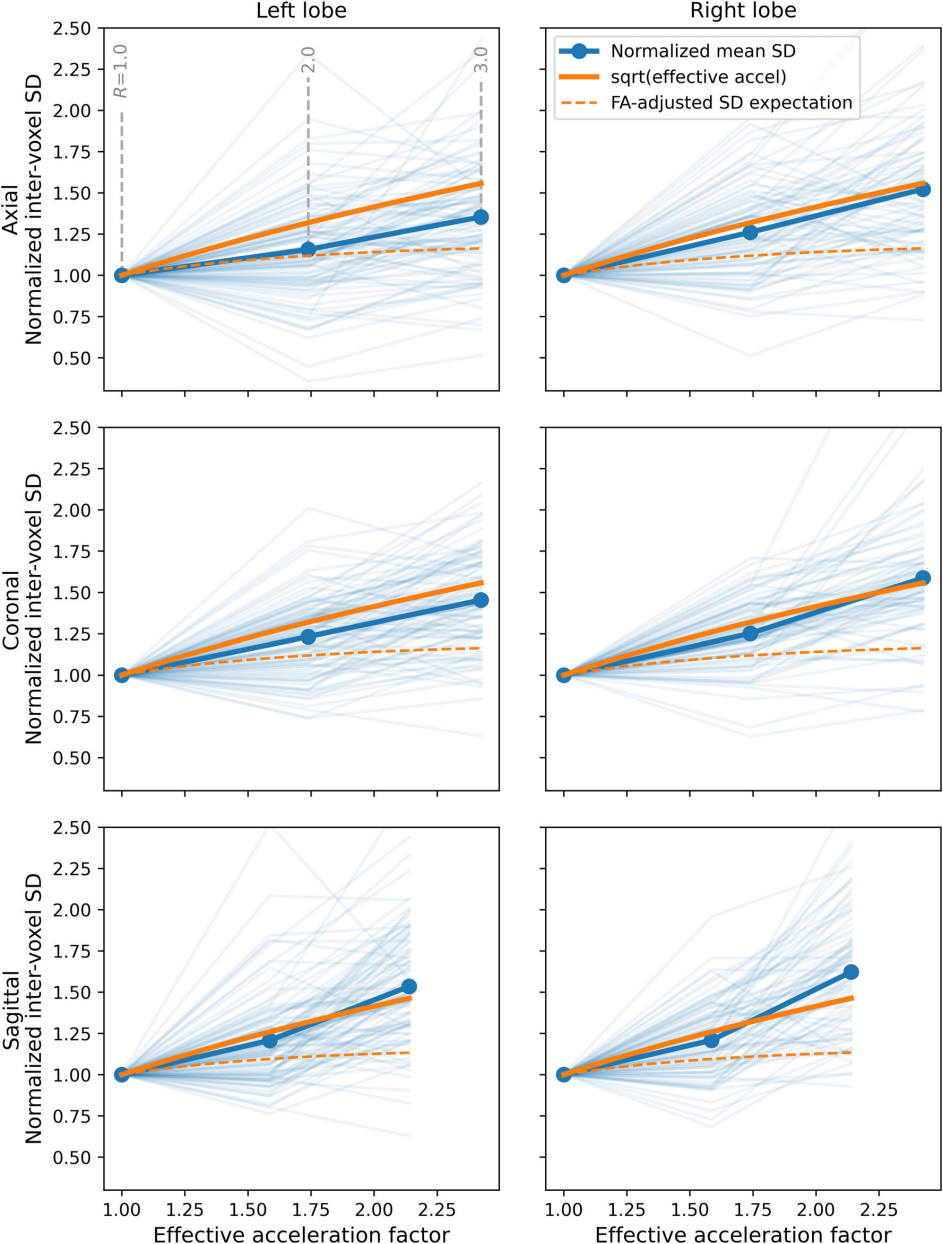
FAM with *R* = 2.0 shows similar or better noise performance for PDFF quantification in vivo than conventionally expected. Small ROIs were drawn on each of the nine Couinaud liver segments in the in vivo acquisitions in this study, and intervoxel SDs were taken as a surrogate for noise. Each segment's SD was normalized to that of the unaccelerated case to track how noise increased as the PI acceleration factor increased (shown in the light blue lines) and plotted here by liver lobe. In both liver lobes and all imaging planes, the average SD for all ROIs (thick blue line) increased slightly slower than the conventionally expected sqrt(effective acceleration factor) relation (thick orange curve) for FAM with *R* = 2.0. This is especially noticeable in the axial plane in the left liver lobe, which experiences the most through‐plane and cardiac motion, and even approaches the theoretical result predicted due to the increase in flip angles (dashed orange curve; see Figure 1 and Theory section).

Finally, FAM with PI acceleration demonstrated lower noise increases, as measured through intervoxel PDFF SDs, than the conventional sqrt(effective acceleration) relation,^18^, ^19^ especially for axial plane imaging at *R* = 2.0 (Figure 9).

## 5 DISCUSSION

In this work, we successfully combined FAM with parallel imaging, optimized the corresponding flip angle schedule, and evaluated its performance in phantoms and in vivo in three orthogonal imaging planes over a range of acceleration factors. Notably, the feasibility of FAM and quantitative performance was demonstrated in vivo in volunteers with a broad range of BMI.

Overall, the reader study demonstrates that FAM is effective in reducing motion artifacts and improving overall image quality, even during free‐breathing, compared to breath‐held 3D‐CSE. The reader study also suggests that axial and coronal FAM acquisitions are superior to sagittal acquisition as measured by image quality and qualitative SNR assessment, whereas sagittal acquisitions are somewhat better for motion artifact reduction. For axial and coronal imaging, acquisitions with *R* = 2.0 were found to be an optimal balance of image quality, qualitative SNR, and motion artifacts.

Quantitative analysis demonstrated that for PDFF and R2*, free‐breathing FAM shows very strong agreement with breath‐held 3D‐CSE. For both PDFF and R2* quantification, the 95% LoA between FAM and 3D‐CSE are similar to the test–retest RC of 3D‐CSE, as reported in this and previous work.^36^ In other words, FAM and 3D‐CSE agree about as well as 3D‐CSE agrees with itself, and the RC of 3D‐CSE could be considered a “lower bound” or “noise floor” for LoA between 3D‐CSE and any proposed new method. Agreement is especially strong in phantoms and in the right lobe of the liver, which experiences less motion, suggesting that FAM shows no bias for PDFF and R2* measured using 3D‐CSE, when 3D‐CSE is less affected by motion artifacts.

In the left lobe of the liver, which experiences more motion, 3D‐CSE shows worse repeatability, illustrating how motion sensitivity degrades the precision of 3D‐CSE. This may also explain the lower agreement between FAM and 3D‐CSE in the left lobe.

The superior test–retest repeatability of coronal FAM in PDFF quantification may be related to reduced through‐plane motion compared to axial FAM because these exams were acquired during free‐breathing, and axial FAM may suffer from greater misregistration due to through‐plane motion. However, although this advantage also applies to sagittal FAM, it did not demonstrate superior repeatability; this may be related to the lower apparent image quality in sagittal FAM, as reflected in the reader study.

The proposed accelerated FAM strategy shows lower noise increases than conventionally expected with increasing PI acceleration, especially at *R* = 2.0. Whereas all FAM methods yield similar quantification results (i.e., signal), accelerated FAM is more SNR‐efficient than unaccelerated FAM because the square root of acquisition time is decreasing faster than SNR is decreasing with PI. This is most readily seen in phantoms, where the noise measurement in phantoms was obtained from voxel‐wise SDs measured across repeated acquisitions without repositioning, so variability across repetitions is attributed solely to noise. In vivo, the noise performance results, as measured through intervoxel SDs, are confounded by the presence of motion and other physiological variability.

Quantitative noise performance results also align with the qualitative SNR ratings from the reader study: There were no statistically significant differences in qualitative SNR ratings between *R* = 1.0 and 2.0 PDFF maps in the axial and coronal planes. To leverage these SNR efficiency gains associated with parallel imaging acceleration, multiple accelerated FAM acquisitions in the coronal or sagittal planes could be obtained. Imaging in these planes would reduce through‐plane motion and ensure whole‐liver coverage. Motion‐corrected averaging could then be performed to realize SNR gains.^37^, ^38^

Multiple other motion‐robust liver fat and iron quantification techniques have been proposed.^9^ These methods can be broadly categorized into non‐Cartesian acquisition strategies, which traverse the center of k‐space repeatedly and average over the respiratory cycle to improve motion robustness, and prospective or retrospective respiratory triggering and motion binning strategies.^9^, ^39^ Non‐Cartesian readouts can also be combined with motion binning and other advanced postprocessing techniques.^40^, ^41^ Compared to non‐Cartesian acquisition strategies, our Cartesian method is less computationally intense to reconstruct, and indeed, an online reconstruction has already been implemented on our system. Non‐Cartesian methods are also generally more prone to errors related to gradient delays, eddy currents, and phase errors.^9^, ^42^, ^43^ However, non‐Cartesian strategies can achieve shorter TEs, which may make them more robust in the case of high R2*. Compared to respiratory triggered or motion binned strategies, one disadvantage of our method is that our data do not naturally form a consistent liver volume, as further discussed below. However, our method is much shorter in acquisition time compared to previous triggered/binned acquisitions (e.g., 80 to 125 s in two previous works^44^, ^45^) and does not rely on any assumptions of quasi‐periodic respiratory motion.

Acquisition time is of practical significance for reducing the cost of CSE‐MRI in clinical translation. Although the reference 3D‐CSE technique used in this study had a shorter acquisition time than FAM, the 3D‐CSE technique is not motion‐robust. Because 3D‐CSE may require rescans due to motion artifacts from inadvertent breathing, it is likely that the FAM technique will reduce total scan time variability and improve clinical workflows compared to 3D‐CSE.^46^, ^47^

There are some limitations with our technique and our study design. With respect to our technique, the current FAM acquisition has relatively long TEs and does not perform well at high R2*, as demonstrated in the PDFF‐R2* phantom. To address this, emerging work has implemented bipolar readouts^48^ (i.e., eliminating flyback gradients) to acquire shorter TEs before the signal decays due to high R2*. The longer TEs in FAM compared to 3D‐CSE may also explain why FAM showed pronounced fat–water swaps more often than 3D‐CSE in the vendor's online reconstruction, which may be calibrated for the shorter TEs.

Further, if FAM is acquired during free‐breathing, the acquired slices may not form a consistent volume, which is a major disadvantage compared to breath‐held acquisitions. This may be addressed through respiratory triggering or registration‐based methods.^37^, ^38^ Assuming one slice is acquired per respiratory cycle (˜4 s), 3D‐CSE and FAM both may take approximately 128 s for full liver coverage (32 slices), similar to previously proposed respiratory‐triggered methods.^44^, ^45^ These acquisition times may be shortened through the use of parallel imaging in the slice direction or simultaneous multi‐slice imaging. Notably, previous work has found respiratory triggering in 3D‐CSE to not significantly improve image quality compared to conventional breath‐held 3D‐CSE.^44^ Additionally, a major advantage of a 2D method such as FAM, unlike 3D‐CSE, is that it can also be split into several breath holds if needed, for example, two ˜13 s breath holds. The advantage of such breath‐held FAM compared to breath‐held 3D‐CSE is that there would be no motion artifacts in the case of inadvertent respiration; however, if the patient can sustain a breath hold, the liver volume imaged would be consistent from slice to slice. We also notice that accelerated FAM is almost short enough for a single breath hold (currently ˜26 s in an axial acquisition, depending on liver size). Adding bipolar readouts,^48^ or other acceleration techniques such as simultaneous multi‐slice acquisitions, could reduce the FAM acquisition to 20 s or faster. This would enable acquisition of all slices within a single breath hold, again with preserved image quality even with inadvertent motion.

Further, bipolar readout would reduce the temporal aperture of each slice, which could potentially be fast enough with a lowered resolution to allow for PDFF and R2* mapping of other organs such as the heart.^49^, ^50^

Finally, in this work FAM was only implemented for a single vendor. However, FAM is based on a relatively simple 2D Cartesian gradient echo sequence, and therefore implementation on other vendors' platforms is likely to be straightforward. Initial evaluation of FAM across vendors has been successfully achieved^51^, ^52^ through the vendor‐agnostic framework Pulseq.^53^, ^54^

With respect to our study, we note some limitations as well, including the use of a small volunteer cohort, although it was sufficient for estimating the bias and limits of agreement of FAM relative to 3D‐CSE with small uncertainties. In addition, the sample size was sufficient for reaching statistically significant results in the reader study. The FAM technique also needs to be further validated in more patients with liver steatosis. Also, the volunteer population represented a wide range of liver PDFF but normal R2*. The limitations of FAM at high R2* require validation in patients with iron overload. Further, this study was conducted at 3.0 T only and further validation at 1.5 T is needed.

## 6 CONCLUSIONS

This study has demonstrated the excellent image quality and quantitative performance of a motion‐insensitive method for liver PDFF quantification, even during free‐breathing. Due to the clinical preference for axial and coronal images, and the low bias, high repeatability, and high image quality of parallel imaging at nominal acceleration factor 2.0, axial or coronal FAM with acceleration 2.0 may be optimal for quantifying PDFF in the liver.

## CONFLICTS OF INTEREST

Dr. Brittain, Dr. Hernando, and Dr. Reeder are co‐founders of Calimetrix, LLC, which manufactured and loaned to the authors the phantoms used in this study. Dr. Brittain and Mr. Kammerman are employees of Calimetrix, LLC. Dr. Reeder is supported by the John H. Juhl Endowed Chair of Radiology. Mr. Tang is a shareholder of GE HealthCare. GE HealthCare provides research support to the University of Wisconsin.

## Supporting information


**FIGURE S1.** Illustration of how the FAM numerical optimization is extended to the autocalibrated k‐space based parallel imaging acceleration case. In the original implementation of FAM by Zhao et al., the lack of parallel imaging means k‐space is sampled uniformly, so the desired signal profile $\vec{f}$ is Gaussian in both the excitations domain and the $k_{y}$ domain. To generalize FAM to the non‐uniform sampling of $k_{y}$ in parallel imaging, we maintain the profile $\vec{f}$ as Gaussian in the $k_{y}$ domain, which means it needs to be non‐Gaussian in the excitations domain. Instead, $\vec{f}$ has a “bend” when the sampling changes from the dense center autocalibration region to the outer accelerated $k_{y}$ regions. For clarity of illustration, this example unaccelerated acquisition has 64 phase‐encode lines; in practical usage, a higher resolution would be acquired. The accelerated acquisition here has 44 phase‐encode lines instead of 64 in the unaccelerated case, representing a temporal aperture reduction of 31%; higher resolution acquisitions would typically see greater proportional reductions at the same nominal acceleration factor.
**FIGURE S2.** Signal profiles produced by FAM are robust to even extreme cases of B1+ inhomogeneity. Previous work has observed B1+ inhomogeneity of 59% to 113% at 3 T (5th to 95th percentile ratios of actual to prescribed flip angles). Here, for FAM in the axial plane with *R* = 2.0, we simulate the effect of scaling the optimized flip angles for this acquisition by 59% and 113%, representing severe cases of B1+ inhomogeneity. Even in such cases, the signal profiles produced are reasonable and likely represent an acceptable form of image filtering.
**FIGURE S3.** Both 3D‐CSE and FAM are accurate in quantifying PDFF at low to moderate R2*, but show artifacts and bias in PDFF quantification at high R2*. A phantom modulated in PDFF (0%–30%) and R2* (50–600 s^−1^) was imaged in 3D‐CSE and FAM, and ROIs were drawn on the vials of the phantom and compared to the nominal PDFF to determine bias. Artifacts can be seen in the 350 and 600 s^−1^ vials in both 3D‐CSE and FAM images. In some configurations of imaging planes and accelerations, FAM shows moderate (˜3%) to severe (˜10%) PDFF bias at high (600 s^−1^) R2*.
**FIGURE S4.** FAM is accurate in R2* quantification at low to moderate R2*, but shows banding artifacts and bias in R2* quantification at high values of R2*. A phantom modulated in PDFF (0%–30%) and R2* (50–600 s^−1^) was imaged in 3D‐CSE and FAM, and ROIs were drawn on the vials of the phantom and compared to the nominal R2* of that vial to determine bias. Banding artifacts can be seen in the 350 and 600 s^−1^ vials in FAM images. FAM generally shows underestimation of R2* at high (600 s^−1^) R2*.
**FIGURE S5.** Reader study results show the strong performance of nominal acceleration factor *R* = 2.0 R2* maps in overall image quality, qualitative SNR, and lack of motion artifacts. Stacked bar plots show the distribution of Likert scale ratings on a subset of R2* maps acquired in this study, pooled from three radiologist readers. Two‐tailed Wilcoxon signed‐rank tests were used in paired comparisons between CSE methods. Blue asterisks to the top left of each stacked bar show significance of comparison with breath‐held (BH) 3D‐CSE. In overall image quality, all free‐breathing (FB) FAM methods other than sagittal with *R* = 3.0 show statistically significant improvement vs. BH 3D‐CSE. In qualitative SNR, axial FAM at all *R* and coronal FAM with *R* = 1.0 had similar or improved SNR vs. 3D‐CSE, while other FAM methods were worse. In the axial and sagittal planes, qualitative SNR did not significantly worsen moving from *R* = 1.0 to 2.0, but did significantly worsen in all imaging planes from *R* = 2.0 to 3.0. In motion artifacts, all FAM methods are significantly better than 3D‐CSE. In each imaging plane, moving from *R* = 1.0 to 2.0 significantly reduced artifacts, but not when moving from *R* = 2.0 to 3.0.
**FIGURE S6.** 3D‐CSE and FAM show especially good agreement in the less mobile right lobe of the liver. The figure shows Bland–Altman analysis of summary PDFF values for various free‐breathing FAM methods compared to the reference, breath‐held 3D‐CSE, with data broken down by liver lobe. Mean differences, 95% limits of agreement (LoAs), and corresponding 95% confidence intervals are given in the plot for each method and liver lobe. FAM generally shows tighter agreement with 3D‐CSE in the right liver lobe, which experiences less cardiac motion. Asterisks indicate significance of differences in LoAs between liver lobes for one particular FAM method. Since 3D‐CSE is more sensitive to motion artifacts, this suggests FAM could be considered as an improved PDFF reference in the more mobile left liver lobe. Different point colors indicate different imaged subjects.
**FIGURE S7.** 3D‐CSE and FAM show strong agreement in vivo for R2* quantification. The figure shows Bland–Altman analysis of R2* summary ROI values for various free‐breathing FAM acquisitions compared to the reference, 3D‐CSE acquired in a breath‐hold. Mean differences, 95% limits of agreement, and corresponding 95% confidence intervals are given in the plot for each method. FAM shows generally good agreement with the 3D‐CSE reference at all accelerations and orientations. The 95% limits of agreement (LoA) between FAM methods and 3D‐CSE is close to the test–retest repeatability of the 3D‐CSE method itself. Different point colors indicate different imaged subjects.
**FIGURE S8.** Free‐breathing (FB) FAM shows similar test–retest repeatability in R2* quantification compared to breath‐held (BH) 3D‐CSE. The figure shows Bland–Altman test–retest repeatability analysis for R2* summary values from the reference 3D‐CSE and various FAM methods. Mean differences, repeatability coefficients, and corresponding 95% confidence intervals are given in the plot for each method. Acquisitions with *R* = 2.0 and 3.0 show similar repeatability as *R* = 1.0 acquisitions, while enabling shorter scans and a shorter per‐slice temporal aperture. Different point colors indicate different imaged subjects.
**FIGURE S9.** Free‐breathing (FB) FAM shows similar repeatability across liver lobes, unlike breath‐held (BH) 3D‐CSE. The figure shows Bland–Altman repeatability analysis for summary PDFF values for the reference 3D‐CSE and FAM methods, broken down by liver lobe. Mean differences, repeatability coefficients, and corresponding 95% confidence intervals are given in the plot for each method and liver lobe. Asterisks indicate significance of differences in RCs between liver lobes for one particular CSE method. 3D‐CSE shows the most dramatic drop in repeatability from the right lobe to the left lobe, although it has the best repeatability in the right lobe. In comparison, FAM shows similar repeatability across liver lobes. Different point colors indicate different imaged subjects.
