# Supplementary material for: Optimized motion‐insensitive PDFF mapping of the liver
Source: Magn Reson Med. 2025 Sep 2;95(1):249–67. doi: 10.1002/mrm.70047 (PMC12490877; doi:10.1002/mrm.70047)
Supplement: Supplementary file 1 — FIGURE S1. Illustration of how the FAM numerical optimization is extended to the autocalibrated k‐space based parallel imaging acceleration case. In the original implementation of FAM by Zhao et al., the lack of parallel imaging means k‐space is sampled uniformly, so the desired signal profile f→ is Gaussian in both the excitations domain and the ky domain. To generalize FAM to the non‐uniform sampling of ky in parallel imaging, we maintain the profile f→ as Gaussian in the ky domain, which means it needs to be non‐Gaussian in the excitations domain. Instead, f→ has a “bend” when the sampling changes from the dense center autocalibration region to the outer accelerated ky regions. For clarity of illustration, this example unaccelerated acquisition has 64 phase‐encode lines; in practical usage, a higher resolution would be acquired. The accelerated acquisition here has 44 phase‐encode lines instead of 64 in the unaccelerated case, representing a temporal aperture reduction of 31%; higher resolution acquisitions would typically see greater proportional reductions at the same nominal acceleration factor. FIGURE S2. Signal profiles produced by FAM are robust to even extreme cases of B1+ inhomogeneity. Previous work has observed B1+ inhomogeneity of 59% to 113% at 3 T (5th to 95th percentile ratios of actual to prescribed flip angles). Here, for FAM in the axial plane with R = 2.0, we simulate the effect of scaling the optimized flip angles for this acquisition by 59% and 113%, representing severe cases of B1+ inhomogeneity. Even in such cases, the signal profiles produced are reasonable and likely represent an acceptable form of image filtering. FIGURE S3. Both 3D‐CSE and FAM are accurate in quantifying PDFF at low to moderate R2*, but show artifacts and bias in PDFF quantification at high R2*. A phantom modulated in PDFF (0%–30%) and R2* (50–600 s−1) was imaged in 3D‐CSE and FAM, and ROIs were drawn on the vials of the phantom and compared to the nominal PDFF to determ [file MRM-95-249-s001.docx]

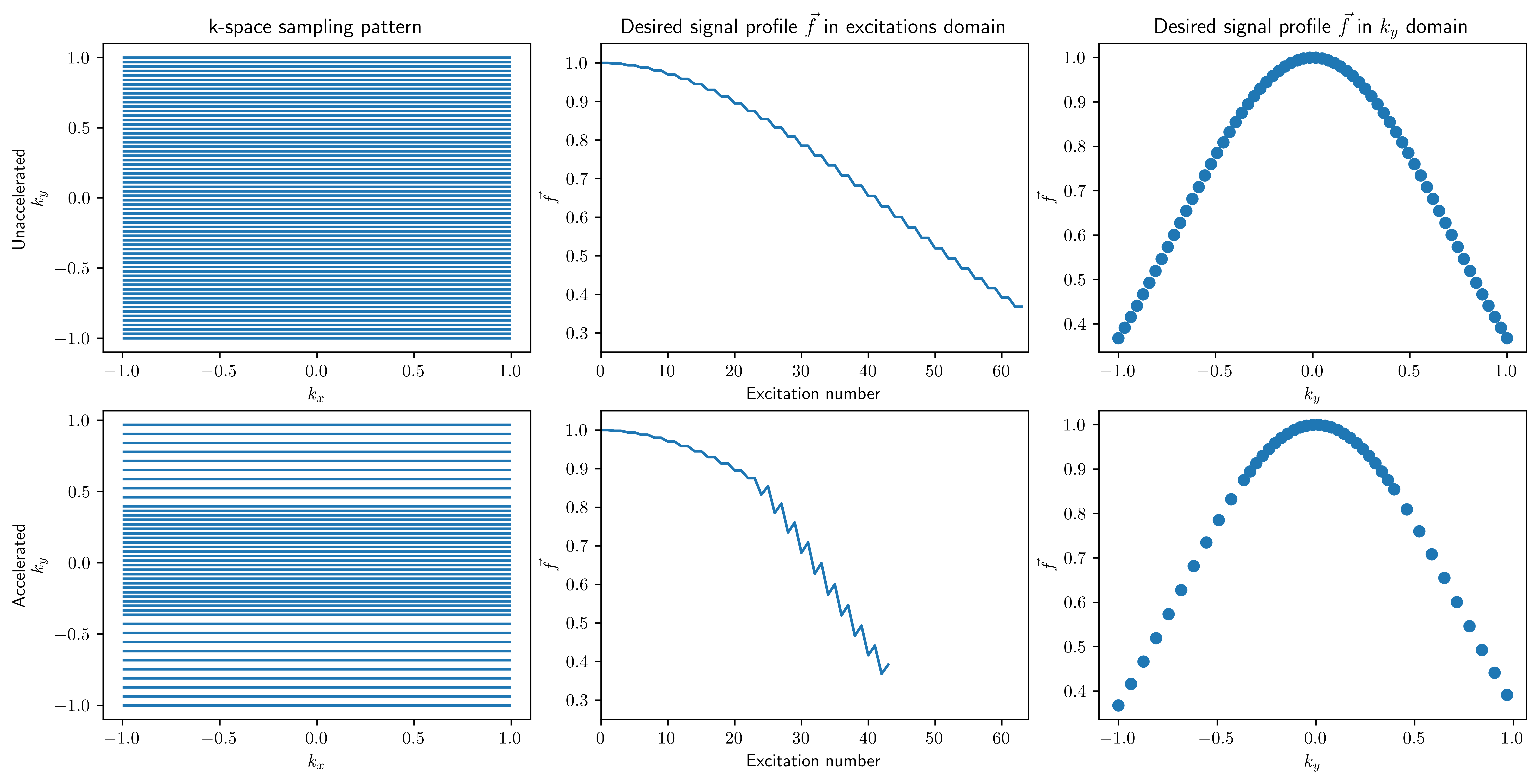


**Figure S1:** Illustration of how the FAM numerical optimization is extended to the autocalibrated k-space based parallel imaging acceleration case. In the original implementation of FAM by Zhao et al., the lack of parallel imaging means k-space is sampled uniformly, so the desired signal profile $\vec{f}$ is Gaussian in both the excitations domain and the $k_{y}$ domain. To generalize FAM to the non-uniform sampling of $k_{y}$ in parallel imaging, we maintain the profile $\vec{f}$ as Gaussian in the $k_{y}$ domain, which means it needs to be non-Gaussian in the excitations domain. Instead, $\vec{f}$ has a “bend” when the sampling changes from the dense center autocalibration region to the outer accelerated $k_{y}$ regions. For clarity of illustration, this example unaccelerated acquisition has 64 phase encode lines; in practical usage, a higher resolution would be acquired. The accelerated acquisition here has 44 phase encode lines instead of 64 in the unaccelerated case, representing a temporal aperture reduction of 31%; higher resolution acquisitions would typically see greater proportional reductions at the same nominal acceleration factor.


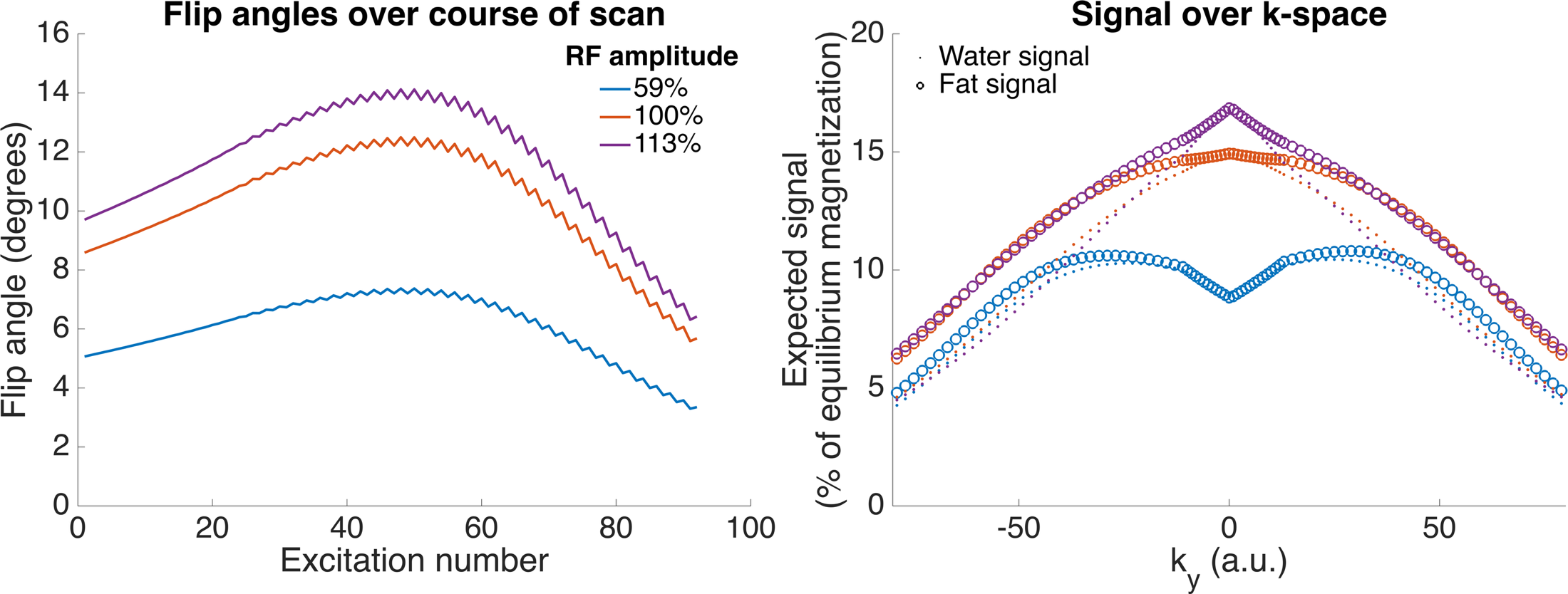


**Figure S2:** Signal profiles produced by FAM are robust to even extreme cases of B1+ inhomogeneity. Previous work has observed B1+ inhomogeneity of 59% to 113% at 3T (5th to 95th percentile ratios of actual to prescribed flip angles). Here, for FAM in the axial plane with *R*=2.0, we simulate the effect of scaling the optimized flip angles for this acquisition by 59% and 113%, representing severe cases of B1+ inhomogeneity. Even in such cases, the signal profiles produced are reasonable and likely represent an acceptable form of image filtering.


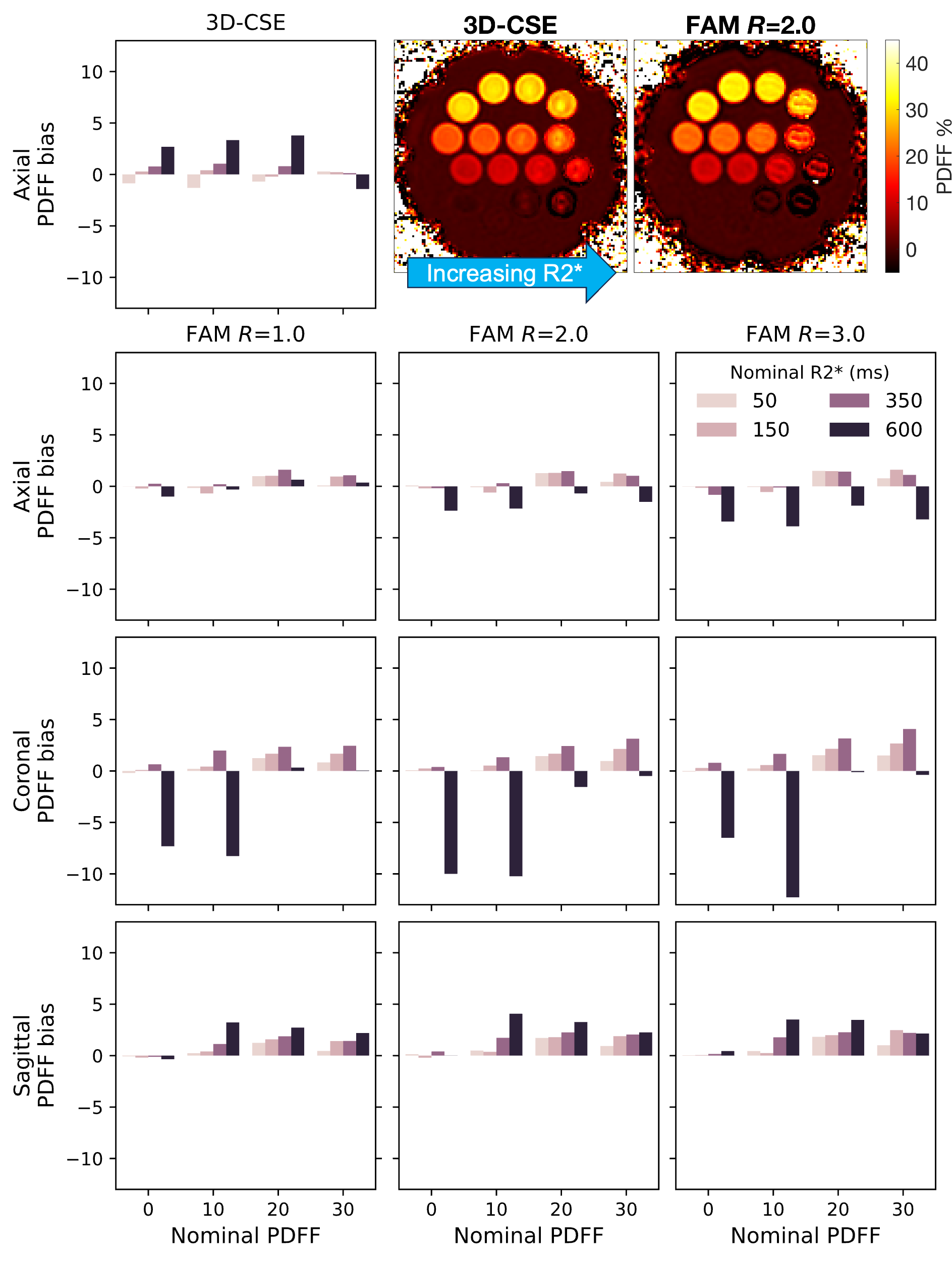


**Figure S3:** Both 3D-CSE and FAM are accurate in quantifying PDFF at low to moderate R2*, but show artifacts and bias in PDFF quantification at high R2*. A phantom modulated in PDFF (0-30%) and R2* (50-600 s^-1^) was imaged in 3D-CSE and FAM, and ROIs were drawn on the vials of the phantom and compared to the nominal PDFF to determine bias. Artifacts can be seen in the 350 and 600 s^-1^ vials in both 3D-CSE and FAM images. In some configurations of imaging planes and accelerations, FAM shows moderate (~3%) to severe (~10%) PDFF bias at high (600 s^-1^) R2*.


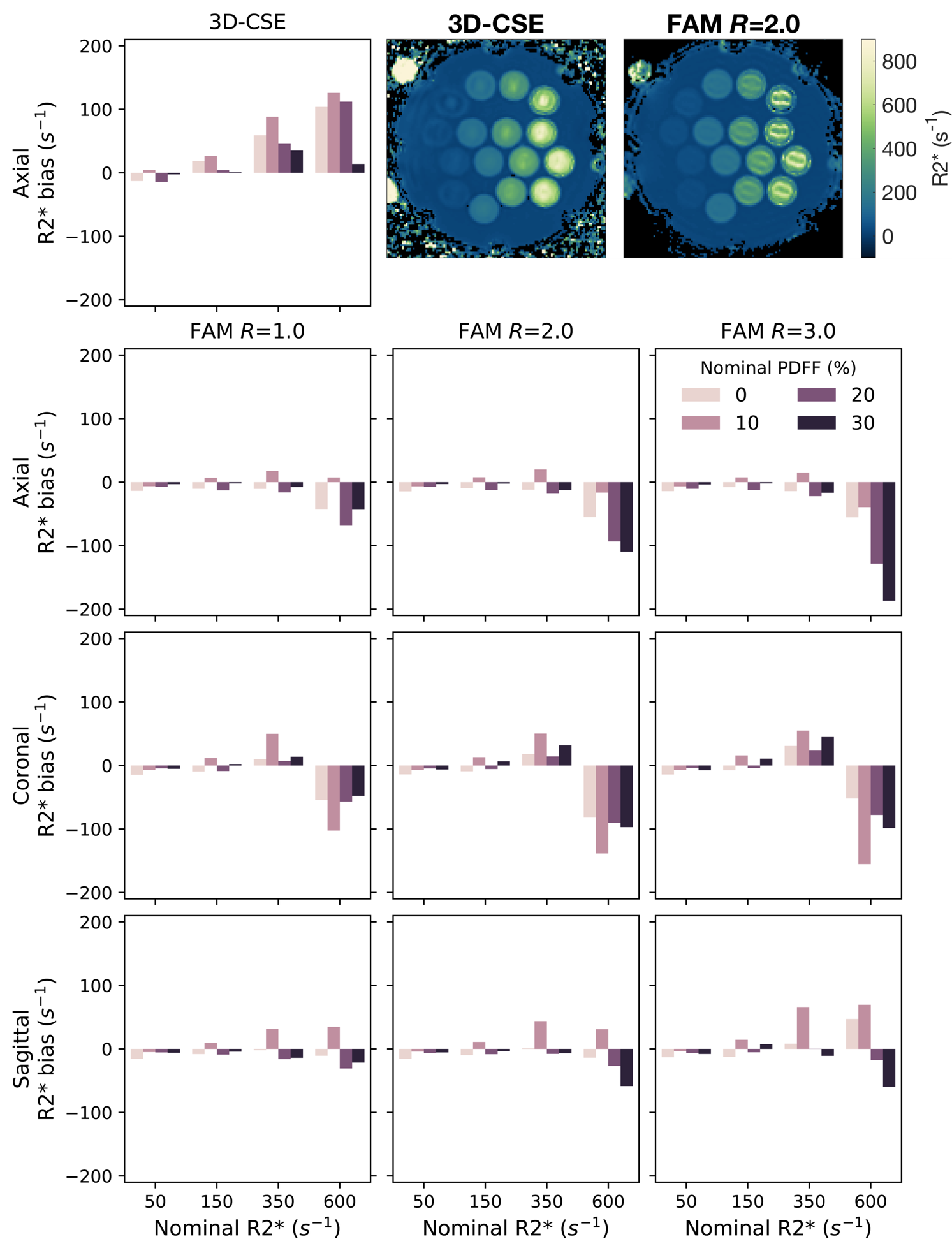


**Figure S4:** FAM is accurate in R2* quantification at low to moderate R2*, but shows banding artifacts and bias in R2* quantification at high values of R2*. A phantom modulated in PDFF (0-30%) and R2* (50-600 s^-1^) was imaged in 3D-CSE and FAM, and ROIs were drawn on the vials of the phantom and compared to the nominal R2* of that vial to determine bias. Banding artifacts can be seen in the 350 and 600 s^-1^ vials in FAM images. FAM generally shows underestimation of R2* at high (600 s^-1^) R2*.


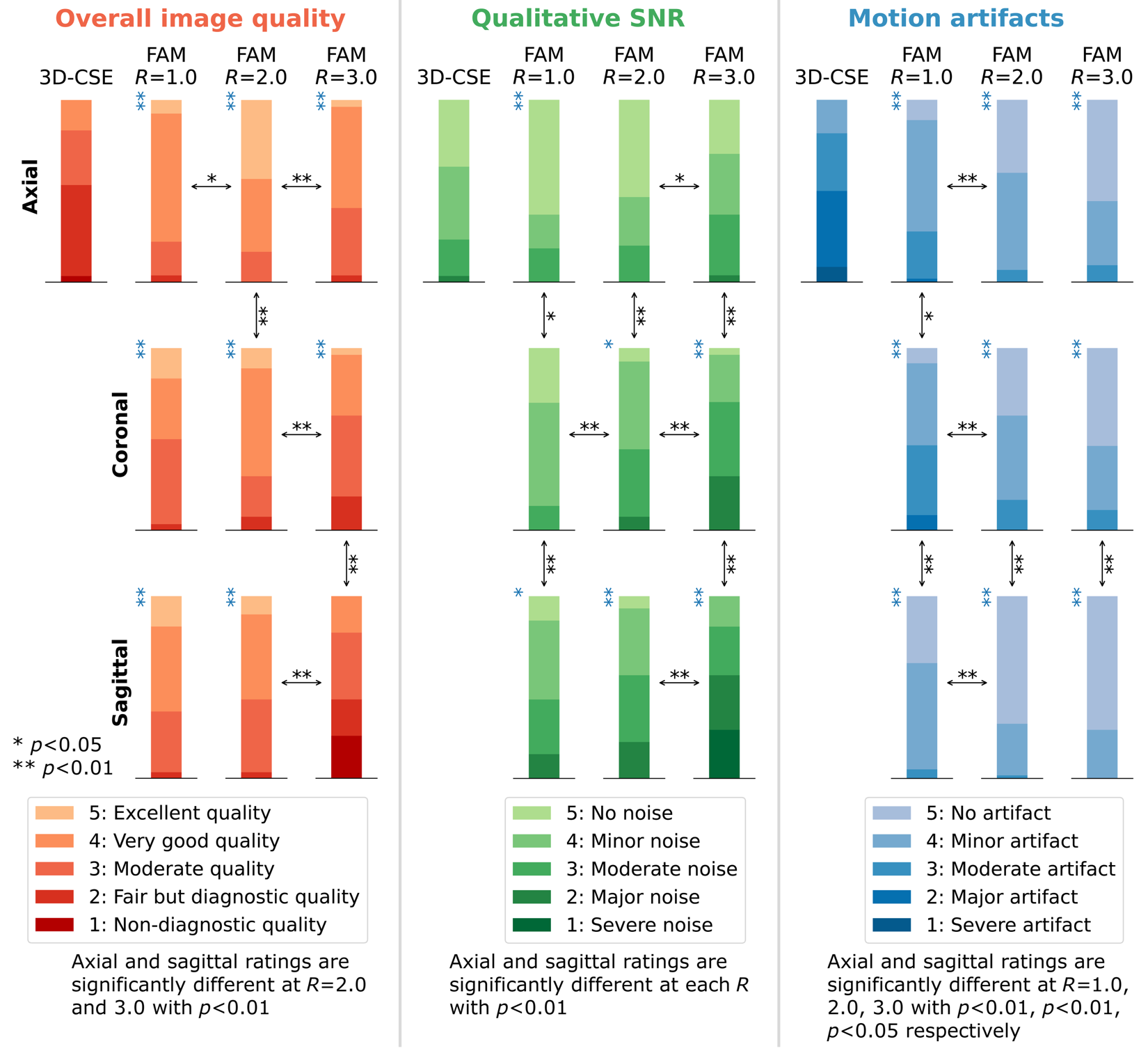


**Figure S5:** Reader study results show the strong performance of nominal acceleration factor *R=*2.0 R2* maps in overall image quality, qualitative SNR, and lack of motion artifacts. Stacked bar plots show the distribution of Likert scale ratings on a subset of R2* maps acquired in this study, pooled from three radiologist readers. Two-tailed Wilcoxon signed-rank tests were used in paired comparisons between CSE methods. Blue asterisks to the top left of each stacked bar show significance of comparison with breath-held (BH) 3D-CSE. In overall image quality, all free-breathing (FB) FAM methods other than sagittal with *R=*3.0 show statistically significant improvement vs. BH 3D-CSE. In qualitative SNR, axial FAM at all *R* and coronal FAM with *R=*1.0 had similar or improved SNR vs. 3D-CSE, while other FAM methods were worse. In the axial and sagittal planes, qualitative SNR did not significantly worsen moving from *R=*1.0 to 2.0, but did significantly worsen in all imaging planes from *R=*2.0 to 3.0. In motion artifacts, all FAM methods are significantly better than 3D-CSE. In each imaging plane, moving from *R=*1.0 to 2.0 significantly reduced artifacts, but not when moving from *R=*2.0 to 3.0.


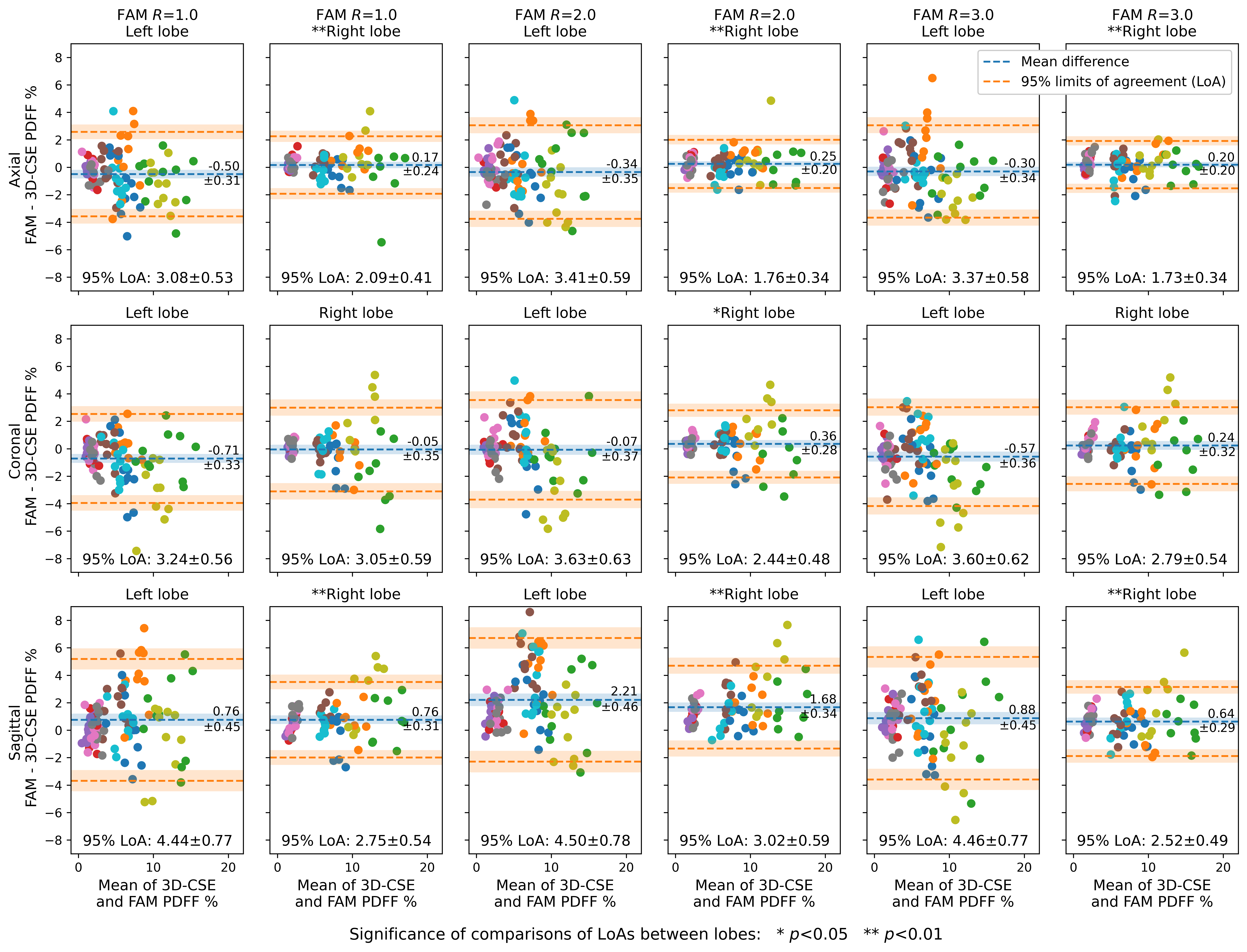


**Figure S6:** 3D-CSE and FAM show especially good agreement in the less mobile right lobe of the liver. The figure shows Bland-Altman analysis of summary PDFF values for various free-breathing FAM methods compared to the reference, breath-held 3D-CSE, with data broken down by liver lobe. Mean differences, 95% limits of agreement (LoAs), and corresponding 95% confidence intervals are given in the plot for each method and liver lobe. FAM generally shows tighter agreement with 3D-CSE in the right liver lobe, which experiences less cardiac motion. Asterisks indicate significance of differences in LoAs between liver lobes for one particular FAM method. Since 3D-CSE is more sensitive to motion artifacts, this suggests FAM could be considered as an improved PDFF reference in the more mobile left liver lobe. Different point colors indicate different imaged subjects.

**
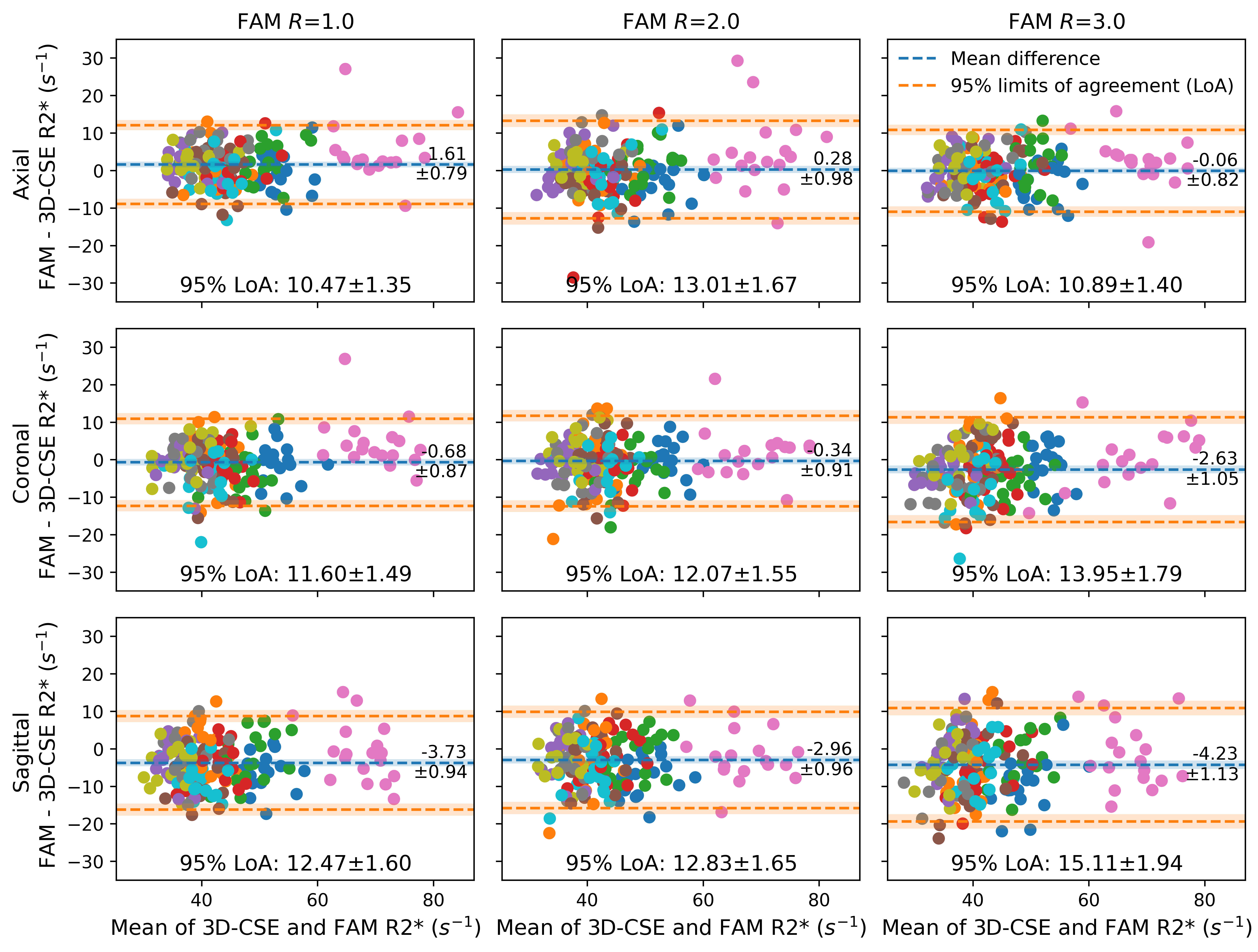
**

**Figure S7:** 3D-CSE and FAM show strong agreement in vivo for R2* quantification. The figure shows Bland-Altman analysis of R2* summary ROI values for various free-breathing FAM acquisitions compared to the reference, 3D-CSE acquired in a breath-hold. Mean differences, 95% limits of agreement, and corresponding 95% confidence intervals are given in the plot for each method. FAM shows generally good agreement with the 3D-CSE reference at all accelerations and orientations. The 95% limits of agreement (LoA) between FAM methods and 3D-CSE is close to the test-retest repeatability of the 3D-CSE method itself. Different point colors indicate different imaged subjects.


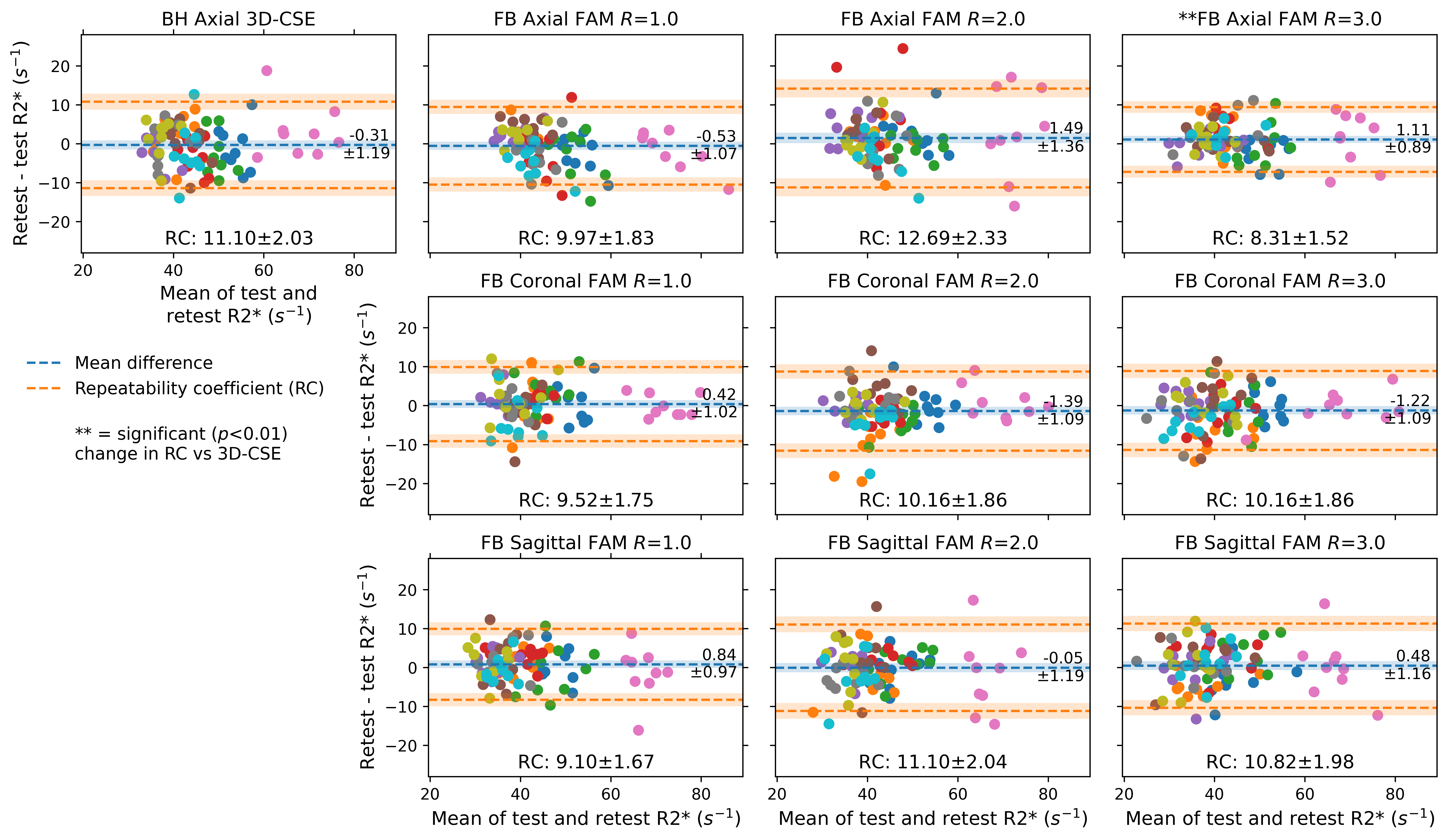


**Figure S8:** Free-breathing (FB) FAM shows similar test-retest repeatability in R2* quantification compared to breath-held (BH) 3D-CSE. The figure shows Bland-Altman test-retest repeatability analysis for R2* summary values from the reference 3D-CSE and various FAM methods. Mean differences, repeatability coefficients, and corresponding 95% confidence intervals are given in the plot for each method. Acquisitions with *R=*2.0 and 3.0 show similar repeatability as *R=*1.0 acquisitions, while enabling shorter scans and a shorter per-slice temporal aperture. Different point colors indicate different imaged subjects.


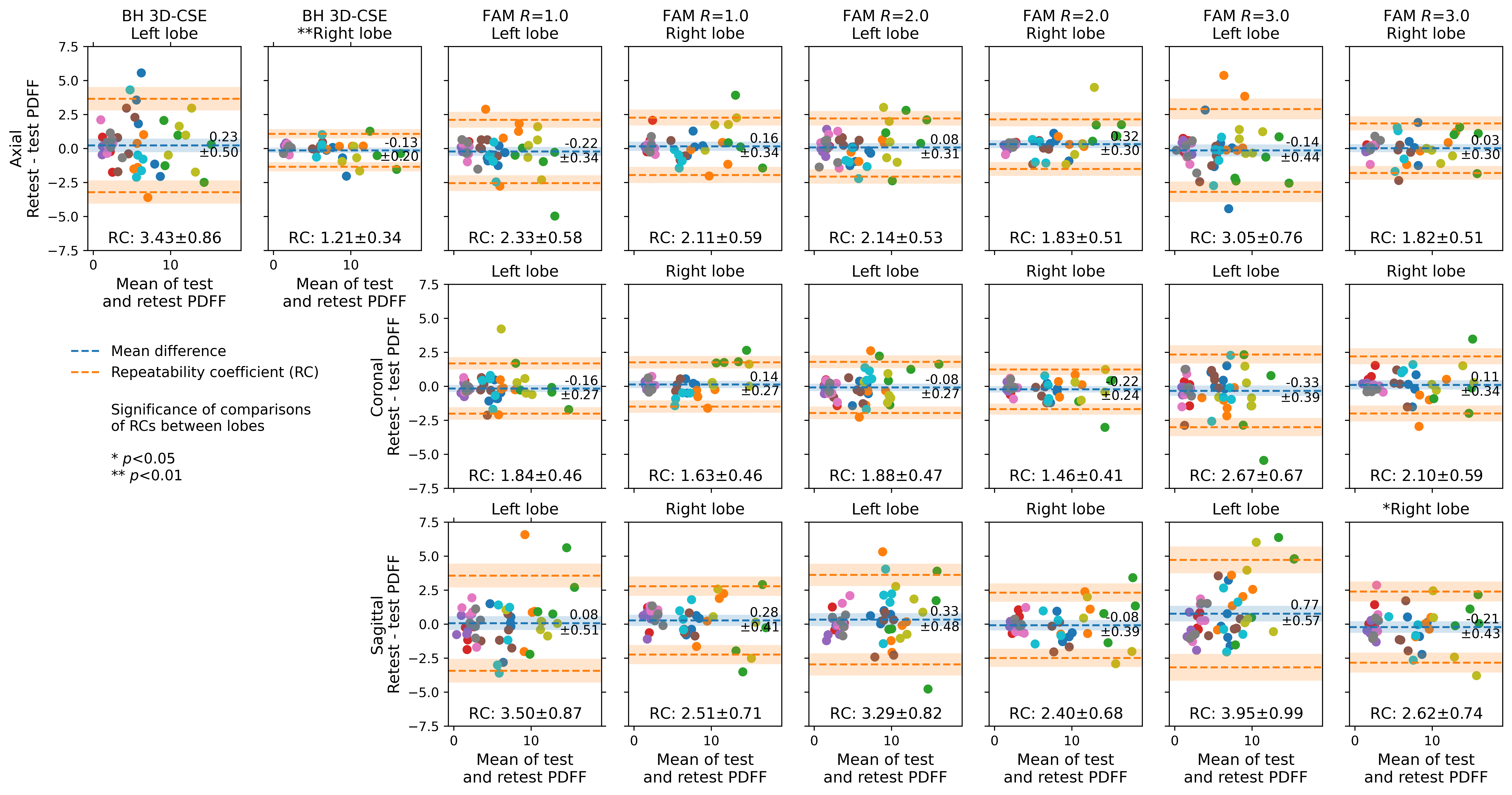


**Figure S9:** Free-breathing (FB) FAM shows similar repeatability across liver lobes, unlike breath-held (BH) 3D-CSE. The figure shows Bland-Altman repeatability analysis for summary PDFF values for the reference 3D-CSE and FAM methods, broken down by liver lobe. Mean differences, repeatability coefficients, and corresponding 95% confidence intervals are given in the plot for each method and liver lobe. Asterisks indicate significance of differences in RCs between liver lobes for one particular CSE method. 3D-CSE shows the most dramatic drop in repeatability from the right lobe to the left lobe, although it has the best repeatability in the right lobe. In comparison, FAM shows similar repeatability across liver lobes. Different point colors indicate different imaged subjects.
